# NKT cells promote Th1 immune bias to dengue virus that governs long-term protective antibody dynamics

**DOI:** 10.1172/JCI169251

**Published:** 2024-08-01

**Authors:** Youngjoo Choi, Wilfried A.A. Saron, Aled O’Neill, Manouri Senanayake, Annelies Wilder-Smith, Abhay P.S. Rathore, Ashley L. St. John

**Affiliations:** 1 Programme in Emerging Infectious Diseases, Duke–National University of Singapore Medical School, Singapore.; 2 Department of Paediatrics, Faculty of Medicine, University of Colombo, Colombo, Sri Lanka.; 3 Lady Ridgeway Children’s Hospital, Colombo, Sri Lanka.; 4 Lee Kong Chian School of Medicine, Nanyang Technological University, Singapore.; 5 Department of Disease Control, London School of Hygiene and Tropical Medicine, London, United Kingdom.; 6 Department of Pathology, Duke University Medical Center, Durham, North Carolina, USA.; 7 Department of Microbiology and Immunology, Yong Loo Lin School of Medicine, National University of Singapore, Singapore.; 8 SingHealth Duke–NUS Global Health Institute, Singapore.

**Keywords:** Immunology, Infectious disease, Cellular immune response, Immunoglobulins, NKT cells

## Abstract

NKT cells are innate-like T cells, recruited to the skin during viral infection, yet their contributions to long-term immune memory to viruses are unclear. We identified granzyme K, a product made by cytotoxic cells including NKT cells, as linked to induction of Th1-associated antibodies during primary dengue virus (DENV) infection in humans. We examined the role of NKT cells in vivo using DENV-infected mice lacking CD1d-dependent (CD1d^dep^) NKT cells. In CD1d-KO mice, Th1-polarized immunity and infection resolution were impaired, which was dependent on intrinsic NKT cell production of IFN-γ, since it was restored by adoptive transfer of WT but not IFN-γ–KO NKT cells. Furthermore, NKT cell deficiency triggered immune bias, resulting in higher levels of Th2-associated IgG1 than Th1-associated IgG2a, which failed to protect against a homologous DENV rechallenge and promoted antibody-dependent enhanced disease during secondary heterologous infections. Similarly, Th2 immunity, typified by a higher IgG4/IgG3 ratio, was associated with worsened human disease severity during secondary infections. Thus, CD1d^dep^ NKT cells establish Th1 polarity during the early innate response to DENV, which promotes infection resolution, memory formation, and long-term protection from secondary homologous and heterologous infections in mice, with consistent associations observed in humans. These observations illustrate how early innate immune responses during primary infections can influence secondary infection outcomes.

## Abstract 2

NKT cells promote long-term protective antibodies and immune memory to dengue virus

## Graphical abstract

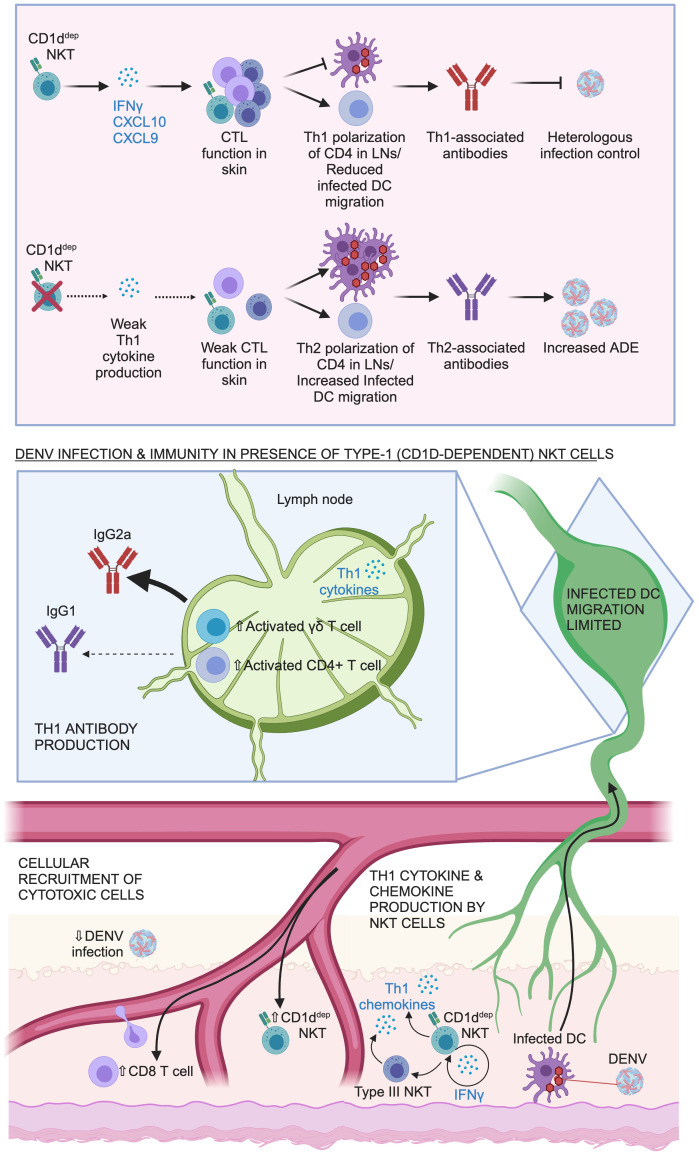

## Introduction

Viral pathogens such as flaviviruses, coronaviruses, and influenza viruses pose unique challenges for adaptive immune responses because of the existence of many serotypes and/or the frequent emergence of new variants. Because of antigenic similarities between the species within these families, reexposure to a heterotypic viral challenge can elicit an adaptive immune response that is partially protective or, alternatively, potentially enhancing for infection or pathogenesis (1). However, early innate immune events are understood to both limit primary viral infection and influence downstream adaptive responses, but how innate immune responses influence memory recall and protection in secondary infections is hardly understood.

One virus for which preexisting heterologous immunity can be problematic is dengue virus (DENV), an arboviral pathogen with a substantial worldwide burden as the most common vector-borne viral disease (2, 3). Having 4 serotypes and a multitude of closely related viruses in the same family, DENV poses a unique public health challenge with respect to its high frequency of reinfection or reexposure to antigenically similar heterotypic infections in the same host, which can alter the disease course (4). Immune memory in DENV infections is associated with strong and long-lasting serotype-specific protection. However, secondary infections with a heterologous serotype of DENV are associated with a risk of heightened disease severity (4). It is thought that this risk of severity is dependent primarily on antibody concentration and specificity (5), where nonneutralizing antibodies can lead to antibody-dependent enhancement (ADE) of infection (5), but the immune factors that promote the development of infection-enhancing antibodies are unclear.

DENV infection begins when the virus is s.c. injected into skin by mosquito bite. Viral particles deposited in the skin initially infect local monocytes and DCs and activate resident innate immune sentinel cells, such as mast cells, which prompt cellular recruitment (4, 6–9). Although DENV is able to disseminate via lymphatic vessels to draining lymph nodes (dLNs) and establish replication in various target organs, this early innate immune response is key for limiting infection (9, 10). Early clearance of DENV from the skin is promoted by an innate response involving innate-like T cells such as γδT cells and NK1.1^+^ cells (6, 11). Both NK and NKT cells are recruited to the skin, with enrichment for NKT cells (6), but it is not known which subsets of NK1.1^+^ T cells are consequential to DENV infection clearance and whether they influence long-term immune memory.

NKT cells display a hybrid transcriptional phenotype of both NK and T cells (12, 13) and can rapidly produce a variety of cytokines and/or acquire cytotoxic functionality upon T cell receptor (TCR) activation (14, 15). Currently, in mice, NKT cells are classified into 3 main subsets: CD1d-dependent (CD1d^dep^) type I (also called iNKT), CD1d-dependent type II, and CD1d-independent (CD1d^ind^) NKT-like cells (15), which differ in their abilities to recognize specific antigens (12, 14, 16, 17) as well as their cytokine secretion profiles and cytotoxic capabilities (17–20). In humans as well, although there are differences in TCR utilization and in vivo distribution, there are also invariant CD1d^dep^ and CD1d^ind^ NKT cells (17). NKT cells are strongly linked with antiviral immunity. For example, NKT markers were detected on a large percentage of virus-specific CD8^+^ and CD4^+^ T cells during lymphocytic choriomeningitis virus infection in mice (21). NKT cell deficiency also led to impaired clearance of herpes simplex and hepatitis B virus infections in mice (22, 23), and NKT cells were shown to be responsible for induction of virus-specific cytotoxic T lymphocyte (CTL) responses against respiratory syncytial and influenza viruses (24, 25). While there is evidence that NKT cells can influence other immune cells through regulating cytokine secretion or interaction with antigen-presenting cells (APCs) (26), the specific contributions of NKT cells (independent of NK cells) to immunity and clearance of cutaneous virus infection are not fully understood. Furthermore, their influence on adaptive immunity and long-term memory responses, and especially those independent of their ability to, themselves, convert to memory phenotypes, have not been adequately explored.

Here, we identified a link between biomarkers of NKT cell activation and a Th1-polarized immune response in primary dengue patients, involving both serum cytokines and antibody subclasses. Using mice deficient in CD1d^dep^ NKT cells, we disentangled the contributions of NKT cells to innate clearance of acute infection from their influence on adaptive responses and learned that NKT cell–driven adaptive immunity can regulate secondary infection outcomes. We observed that NKT-dependent Th1-immune polarization in mice led to protection during secondary homologous and heterologous infections in a mechanism dependent on the intrinsic production of IFN-γ by NKT cells. As in mice, severe disease in secondary dengue patients was strongly associated with a Th2 characteristic profile of preexisting antibodies. These results support a dual role for CD1d^dep^ NKT cells in DENV infection resolution dependent on Th1-associated immune responses as well as long-term antibody-mediated protection against reinfection.

## Results

### NKT cell activity primes for Th1 immune bias during primary human dengue infections.

**Figure 1:**
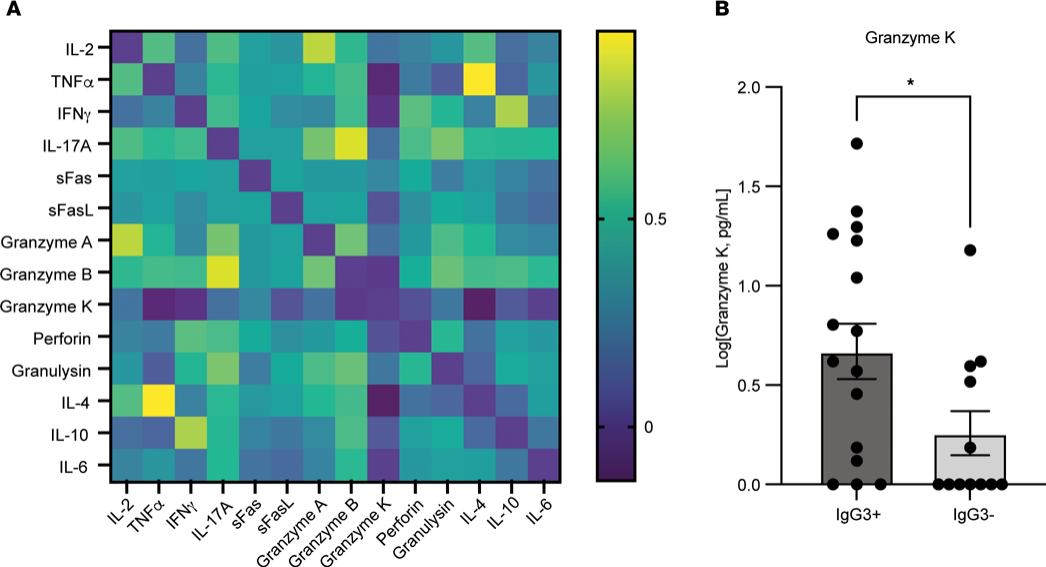
Association of serum granzyme K concentrations with subsequent IgG3 induction in primary dengue patients. Concentrations of cytokines and T cell–associated immune factors were determined in the serum of pediatric primary dengue patients (*n* = 29). (**A**) Correlation matrix showing the correlation between concentrations of cytokines in patient samples at the acute (days 1–3 after fever onset) phase of disease. Heatmap represents the correlation coefficients for each pair of cytokines evaluated. Stronger positive correlations approach 1. (**B**) Serum concentrations of granzyme K were significantly higher in serum samples obtained 1 to 3 days after fever onset for patients who had detectable anti-DENV IgG3 by days 6 to 7 after fever onset. *P = 0.0495, Mann Whitney *U* test. IgG3 positivity was defined as having an anti-DENV endpoint ELISA titer 2-fold or greater above that of naive serum.

We first questioned whether immune signatures of cytotoxic lymphocyte activation could be a predictor of efficient induction of Th1-associated immune responses in DENV-infected humans. Human dengue-confirmed patients from a hospitalized pediatric cohort were longitudinally sampled once between days 1 and 3 and again between days 6 and 7 after fever onset, representing time points of acute infection (i.e., at the time of patient presentation at the clinic and recruitment) and the beginning of adaptive immune responses, respectively (4, 27). For the primary dengue patients, biomarkers associated with the functions of cytotoxic lymphocytes were enriched in the serum of some patients in early acute infection (1 to 3 days after fever onset) (Figure 1A). Furthermore, there were strong correlations between the levels of granzyme A and IL-2, granzyme B and IL-17, and IL-4 and TNF in patient sera (Figure 1A), which suggests clustering of patients whose early immune profiles were typified by Th1-, Th17-, or Th2-type responses. Levels of IL-10 and IFN-γ, which have previously been shown to be produced by CD4^+^ T cells and linked to dengue disease (28), also appeared correlated with each other (Figure 1A). We next questioned whether the serum levels of any of these cytokines were associated with the early induction of a Th1-polarized antibody response. Interestingly, the levels of granzyme K during the acute phase of infection were significantly higher in patients who displayed effective class-switch of DENV-specific antibodies to the Th1-associated isotype IgG3, which was measured in the defervescence phase of disease in longitudinal samples (Figure 1B). DENV-specific IgG4, which is a Th2-associated human antibody subclass (29), could not be detected, which is consistent with literature suggesting that it takes several weeks for IgG4 induction following primary DENV infection (30), while IgG2 was only detected in 14.3% of patients (Supplemental Figure 1A; supplemental material available online with this article; https://doi.org/10.1172/JCI169251DS1). These IgG3 and IgG4 antibody subclasses are usually low abundance in human serum compared with IgG1/2 and, aside from being associated with Th1/2 polarization, can illustrate a refined antibody response following germinal center activity and effective class-switching (31). In contrast, IgG1, the most abundant subclass of serum antibodies in humans (31), was produced by 97% of patients by this time point after fever onset, but did not correlate with granzyme K (Supplemental Figure 1, B and C). In spite of its association with Th1-polarized IgG3 antibody responses, granzyme K was not associated with serum virus burden or disease severity during primary infection (Supplemental Figure 1, D and E). Granzyme K is produced primarily by NKT cells, although to a lesser extent by γδT cells, but not made appreciably by traditional CTLs (32). Even though granzyme K is not cell type specific, its strong association with NKT cells raised the question of whether early NKT cell responses could influence the mounting adaptive immune response and Th1 balance during DENV infection.

### CD1d^dep^ NKT cell deficiency alters cellularity of DENV-infected skin and dLNs.

**Figure 2:**
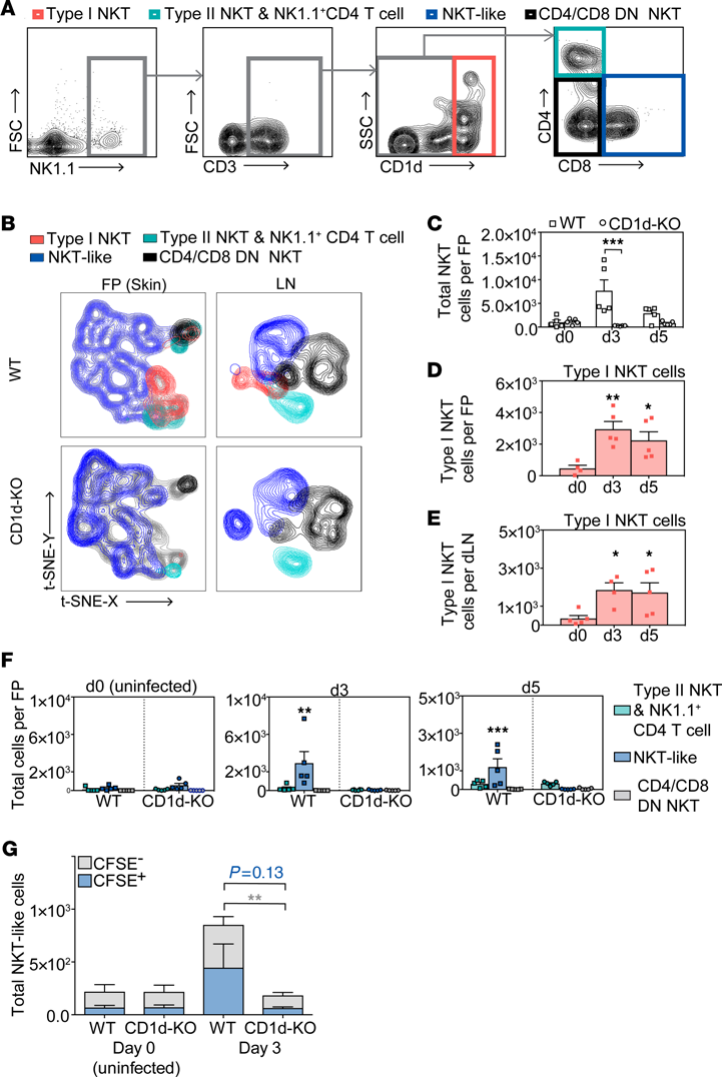
Type I NKT and NKT-like cells are recruited to DENV-infected skin and dLNs. (**A**) Various NKT cell subsets from the skin and dLNs were identified by flow cytometry using CD1d tetramer staining and additional markers, including iNKT (NK1.1^+^CD3^+^CD1d-tetramer^+^), type II NKT cells (NK1.1^+^CD3^+^CD1d-tetramer^–^CD4^+^, which have a phenotype similar to that of NK1.1^+^CD4^+^ T cells), NKT-like NKT cells (NK1.1^+^CD3^+^CD1d-tetramer^–^CD8^+^), and CD1d-tetramer^–^ CD4 CD8 DN NKT cells (NK1.1^+^CD3^+^CD1d-tetramer^–^CD4^–^CD8^–^). FSC, forward scatter; SSC, side scatter. (**B**) The unbiased clustering tSNE algorithm was used to verify flow cytometry data in the FP skin and dLNs. The algorithm was run on concatenated total samples (infected and uninfected). iNKT cells were absent in CD1d-KO mice. (**C**) Total NKT cells were quantified in the skin in WT and CD1d-KO mice after infection with 2 × 10^5^ PFU of DENV2 s.c. by FP injection. DENV infection increased iNKT cells in WT but not in CD1d-KO mice in the (**D**) skin and (**E**) dLNs on days 3 and 5 after infection. (**F**) Quantification of CD1d^ind^ NKT cell subsets in the FP showed increased NKT-like cells at days 3 and 5 after infection in WT but not CD1d-KO mice, while other NKT cell subsets measured were not influenced. (**G**) Tracking of local versus recruited NKT-like cells in the skin, performed by CFSE labeling prior to DENV2 infection. CFSE^+^ or CFSE^–^ NKT-like cell (NK1.1^+^CD3^+^CD1d-tetramer^–^CD8^+^) numbers in the FP skin were determined on day 3 after infection and are presented as stacked bars. iNKT and NKT-like cells were recruited into the skin during DENV infection, and both subsets were absent from the skin of CD1d-KO mice. Data are represented as means ± SEM and are for *n* = 5 mice combined from 2 independent experiments for each time point. *P < 0.05; **P < 0.01; ***P < 0.001, 1- or 2-way ANOVA with Holm-Šidák post test.

Prior to assessing the functional contributions of NKT cells to virus clearance and adaptive responses, we characterized the NKT cell response at the cutaneous site of DENV infection and the dLN, where immunity is initiated, in a well-established immune-competent mouse model of DENV infection (6, 11, 33–36). Since mosquitoes inject DENV particles s.c. during their feeding (37) and because the mouse footpad (FP) skin drains to a single dLN, the popliteal LN (38), we chose this route of infection to examine NKT cell responses. DENV is also a lymphotropic pathogen, and the dLN is the secondary site of infection (4, 9). First, we infected WT and CD1d-KO mice with DENV2 (2 × 10^5^ PFU) via s.c. injection and determined the relative NKT cell subtypes present in the FP skin and dLN by flow cytometry at days 3 and 5 after infection (Figure 2A), which were defined based on cellular markers identified in prior reports (13, 15, 39, 40). A CD1d-tetramer loaded with PBS-57, an analogue of αGAlCer, was used to identify type I, iNKT cells, while type II NKT cells that are also CD1d restricted would not bind to the αGAlCer-loaded tetramer. An unbiased multidimensional reductional analysis (t-sne) was used to visualize and verify the phenotypic differences in NKT cell populations between WT and CD1d-KO mice in the skin and dLNs (Figure 2B). This confirmed the expected absence of iNKT cells (NK1.1^+^CD3^+^CD1d-tetramer^+^) in the skin and dLNs (Figure 2B). In the FP skin of WT mice, we detected NKT-like cells (NK1.1^+^CD3^+^CD1d-tetramer^–^CD8^+^), with very small numbers of type II NKT cells (or CD4^+^ T cells that had acquired the NK1.1 marker, which are phenotypically indistinguishable from NKT cells) (NK1.1^+^CD3^+^CD1d-tetramer^–^CD4^+^) and CD4/CD8 double-negative (CD4/CD8 DN) NKT cells (NK1.1^+^CD3^+^CD1d-tetramer^–^CD4^–^CD8^–^) (Figure 2B). In WT mice, the CD4/CD8 DN NKT population represents a combination of some type II NKT cells and NKT-like cells, although based on the CD1d restriction of type II NKT cells, the same phenotype should represent only NKT-like cells in CD1d-KO mice. As expected, CD1d-KO mice lacked iNKT and type II NKT cells (Figure 2B), and their small residual NK1.1^+^CD4^+^ population should represent T cells that have acquired the NK1.1 marker, which can occur during viral infection (21), while their CD4/CD8 DN population of NK1.1^+^CD3^+^ cells should represent NKT-like cells only. Thus, CD1d^ind^ NKT-like cells were the dominant cell population with NKT cell markers in CD1d-KO mice at baseline (Figure 2B).

After s.c. infection with DENV, total NKT cells were enumerated in the skin and dLNs. In contrast to NK cells, which did not differ between WT and CD1d-KO mice (Supplemental Figure 2, A and B), there were reduced NKT cells in the skin of CD1d-KO mice compared with WT mice on days 3 and 5 after infection (Figure 2C). However, the total NKT cells did not differ in the dLNs between WT and CD1d-KO mice at the same time points of this experiment (Supplemental Figure 2C). In infected WT mice, we also detected a significant increase in iNKT cells in both the FPs and LNs on days 3 and 5 after infection (Figure 2, D and E). Furthermore, significantly increased numbers of NKT-like cells were present on days 3 and 5 after infection in the FPs but not dLNs of WT mice, but not in CD1d-KO mice (Figure 2F and Supplemental Figure 2D). This was confirmed by confocal microscopy, where CD3^+^ or NK1.1^+^ single-positive cells could be identified in the infected skin of CD1d-KO mice, but no double-positive NKT cells could be found, in contrast with WT skin where they were rare, but present (Supplemental Figure 2E). These results suggest that although NKT-like cells are present in CD1d-KO mice, their recruitment to DENV-infected skin is regulated by CD1d^dep^ NKT cells, which are absent in this model, and they are not replenished in the skin following infection. To further validate defects in the recruitment of immune cells to the site of cutaneous DENV infection in the absence of CD1d^dep^ NKT cells, we used CFSE labeling of cells in the FP skin prior to infection to label resident cells (CFSE^+^). We observed significantly higher numbers of newly recruited, CFSE^–^ NKT-like cells in the skin of WT mice compared with CD1d-KO mice on day 3 after infection (Figure 2G). However, we did not detect a significant difference in the number of tissue-resident, CFSE^+^ NKT-like cells in the skin of WT and CD1d-KO mice (Figure 2G). Therefore, the increase in CD1d^ind^ NKT-like cells observed in the skin during DENV infection was primarily due to newly recruited cells. Together, these data demonstrate that CD1d^dep^ NKT cells are necessary for the trafficking of CD1d^ind^ NKT-like cells into the cutaneous site of DENV infection.

In the dLNs, NKT cell phenotypes were similarly analyzed, and we detected diverse subsets of NKT cells and an increase in CD1d^ind^ NKT cell subsets in both WT and CD1d-KO mice following DENV2 infection (Supplemental Figure 2D). In contrast to observations made in the skin, CD1d^ind^ NKT-like cells increased to comparable numbers in the dLNs of CD1d-KO mice compared with WT mice and the numbers of CD4/CD8 DN NKT cells were also increased, but not significantly different between WT and CD1d-KO mice (Supplemental Figure 2D). We also detected increased numbers of NK1.1^+^CD3^+^CD4^+^CD1d-tetramer^–^ cells in CD1d-KO mice, (Supplemental Figure 2D), which are likely CD4^+^ T cells that acquired the NK1.1 marker, as conventional CD4^+^ and CD8^+^ T cells have been shown to express NK1.1 upon acute viral infection (21). Similar to the skin (Supplemental Figure 2E), NKT cells could be identified visually in the infected LNs of WT mice, but these were not abundant in dLNs of CD1d-KO mice (Supplemental Figure 2, F and G). These results support that, unlike the skin site of infection where recruitment of CD1d^ind^ NKT cell types was affected by CD1d deficiency, the cellularity of the dLNs with regards to NKT cell subsets was not significantly affected, aside from the confirmed lack of CD1d^dep^ NKT cells.

**Figure 3:**
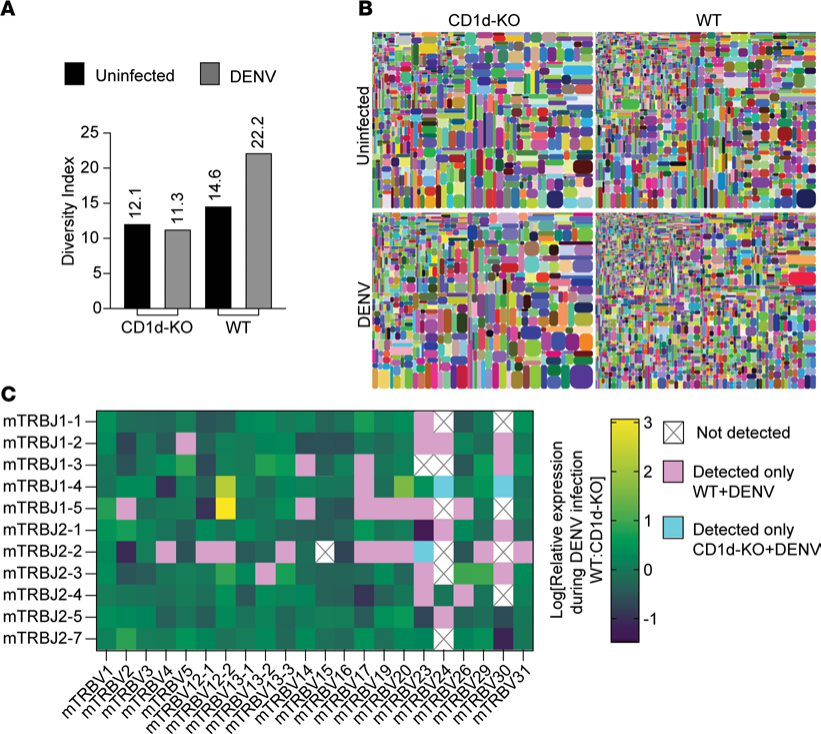
Reduced diversity of NKT cell TCR clones in LNs of DENV-infected CD1d-KO mice. CD3^+^NK1.1^+^ T cells from mouse dLNs (mesenteric and iliac) collected and pooled from *n* = 3 mice per group were harvested and sorted 72 hours after i.p. infection with DENV. The TCR-β region was sequenced to identify the abundance of unique clones. (**A**) Diversity index calculated by iRepertoire software iRweb, version 3.0.1, which reflects the diversity of unique CDR3s clones sequenced for each sample. (**B**) Tree map illustration of diversity for the respective samples. (**C**) Heatmap of the relative frequencies of given V gene and J gene combinations. The clones uniquely identified in WT and CD1d-KO mice are overlaid in pink and blue, respectively.

To further characterize the subsets of NKT cells in the LNs following infection in WT and CD1d-KO mice, we sorted NK1.1^+^CD3^+^ cells for TCR sequencing. The sequencing results, indicating an increase in TCR diversity in WT but not CD1d-KO mice upon infection, shown by diversity index and visually by tree map presentation of the individual clones (Figure 3, A and B), supported the interpretation that diverse NKT-like cells were recruited in the presence of CD1d-restricted NKT cells. A more in-depth analysis of the V and J pairs showed that certain clones were highly enriched in WT animals, such as pairings of mTRBV12-2 with mTRBJ1-5 or mTRBJ1-4, in addition to the broad increase in frequencies of multiple diverse TCR-β clones (Figure 3C). There were many clones that were only detected in the LN of WT mice during DENV infection and fewer that were only detected in infected CD1d-KO mice (Figure 3C). This might also be consistent with the acquisition of NK1.1 as a marker on conventional T cells in CD1d-KO mice, although there were only 3 clones that were uniquely identified in CD1d-KO mice upon infection (Figure 3C), suggesting the potential of this to occur would be very limited. Together these data emphasize the role of CD1d^dep^ NKT cells, which are invariant, in promoting NKT cells or NKT-like cells with diverse repertoires during infection.

### CD1d^dep^ NKT cells enhance CTL recruitment and promote resolution of infection.

**Figure 4:**
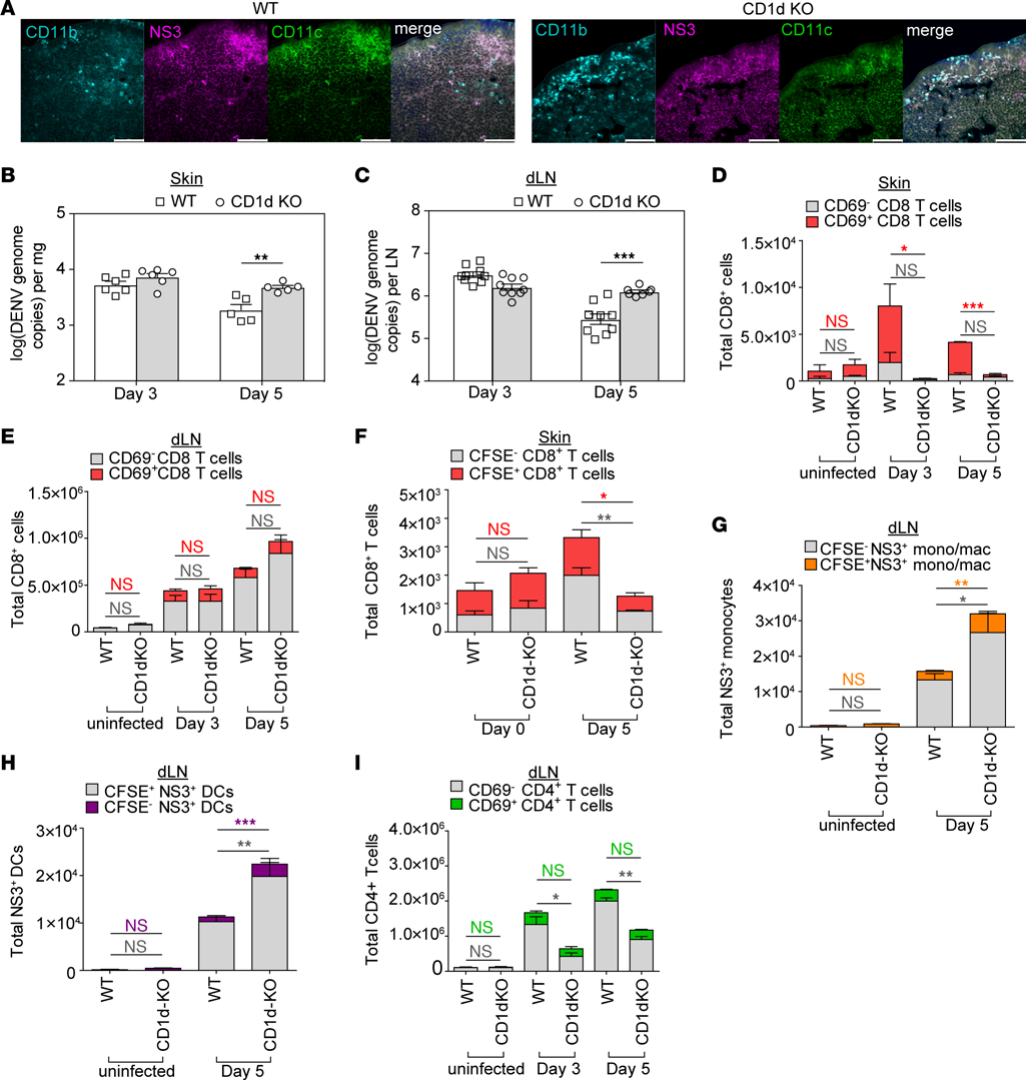
CD1d^dep^ NKT cells accelerate DENV clearance and promote CD8^+^ T cell recruitment and activation in DENV-infected skin. WT and CD1d-KO mice were infected with 2 × 10^5^ PFU of DENV2 s.c. by FP injection. (**A**) DENV NS3 staining of infected cells including DCs and monocyte/macrophages at day 3 in the dLNs of WT and CD1d-KO mice. Scale bars: 50 μM. Additional images are provided in Supplemental Figure 2. (**B** and **C**) DENV2 (E protein) gene copy numbers in the (**B**) FP skin and (**C**) dLNs were determined on days 3 and 5 after infection by qPCR. Single-cell suspensions from FPs and dLNs were stained with antibodies against CD3, CD4, CD8, γδTCR, and CD69 for characterization of the T cell response by flow cytometry. Total and activated CD8^+^ CTL populations in the (**D**) FP skin and (**E**) dLNs were determined on days 0, 3, and 5 after infection. Tracking of local versus recruited cells was performed by CFSE labeling prior to DENV2 infection. (**F**) CFSE^+^ (red) or CFSE^–^ (gray) CD8^+^ T cell (NK1.1^–^CD3^+^CD8^+^) populations in the FPs were determined on day 3 after infection. To track infected cell migration, CFSE was injected into the FPs of WT and CD1d-KO mice 3 days after DENV2 infection. (**G**) DENV2-infected monocytes (mono) (CD45^+^CD11b^+^CD11c^–^MHCII^+^DENV-NS3^+^CFSE^–^; gray) and FP-derived, DENV2-infected monocyte/macrophage (mac) (CD45^+^CD11b^+^CD11c^–^MHCII^+^DENV-NS3^+^CFSE^+^; orange) numbers in the dLNs were determined 5 days after infection. (**H**) DENV2-infected DCs (CD45^+^CD11b^–^CD11c^+^MHCII^+^DENV-NS3^+^CFSE^–^; gray) and FP-derived, DENV2-infected DCs (CD45^+^CD11b^–^CD11c^+^MHCII^+^DENV-NS3^+^CFSE^+^; purple) in dLNs were enumerated 5 days after infection. (**I**) Total and activated CD4^+^ T cells in dLNs were determined on days 0, 3, and 5 after infection. For all panels, *n* = 6 mice for each time point. Data are represented as means ± SEM. *P < 0.05; **P < 0.01; ***P < 0.001, 2-way ANOVA with Tukey’s post test. For **D**–**I**, stacked bars are presented showing CFSE^+^ and CFSE^–^ populations. CD1d^dep^ NKT cells promote activation of CD8^+^ T cells in DENV-infected skin and clearance of DENV from skin and dLNs.

Next, to examine how CD1d^dep^ NKT cells affect virus clearance, we measured the infection burden at the site of infection, the FP skin, and in the dLNs of WT and CD1d-KO mice on days 3 and 5 after DENV2 infection. Staining of dLNs for monocyte and DC markers CD11b and CD11c revealed that most of the infected cells that were increased were myeloid lineage cells that are known DENV infection targets (Figure 4A). The differences in viral genome copies were not significantly different in the FP and dLNs at day 3 after infection between WT and CD1d-KO mice (Figure 4, B and C). By day 5, higher DENV2 infection levels were detected in the skin (Figure 4B) and dLNs (Figure 4C) of CD1d-KO mice compared with WT mice. This increased burden in the dLNs was confirmed on day 5 by IHC (Supplemental Figure 3). These results suggest that CD1d^dep^ NKT cells promote the resolution of DENV infection and that their absence leads to delayed viral clearance.

To identify the aspects of cellular immunity that could mediate delayed viral clearance in the context of CD1d^dep^ NKT cell deficiency, we examined other conventional T cell responses associated with viral clearance, including those of CD8^+^ CTLs. Indeed, delayed DENV2 clearance in the skin of CD1d-KO mice was associated with a reduced CTL response, as there were fewer total and activated CD8^+^ T cells in the skin of CD1d-KO mice at days 3 and 5 after infection (Figure 4D). In contrast to skin, similar numbers of total and activated CD8^+^ T cells were detected in the dLNs of WT and CD1d-KO mice (Figure 4E). We also quantified tissue-resident or newly recruited CD8^+^ T cells in the FP skin at day 3 after infection to determine whether the reduction in CTL numbers in CD1d-KO skin was due to impaired recruitment and/or in situ proliferation of CD8^+^ T cells. We observed significantly higher numbers of both newly recruited CFSE^–^CD8^+^ T cells and tissue-resident CFSE^+^CD8^+^ T cells in the FPs of WT mice compared with CD1d-KO mice on day 3 after infection, yet no changes at baseline (Figure 4F). These labeling experiments support that the increase in CD8^+^ T cells in the skin during DENV infection is due to both new recruitment and in situ proliferation of CD8^+^ T cells and that CD1d^dep^ NKT cells are necessary for the trafficking of CD8^+^ CTLs within DENV-infected skin and their expansion.

The higher viral load in the dLNs of CD1d-KO mice despite the unaltered CTL response at that site suggested other mechanisms leading to the amplification of DENV infection in the dLNs might exist, aside from merely impaired CTL-mediated clearance of infection. As DENV is lymphotropic and infection-bearing DCs and inflammatory monocyte-derived cells migrate from the cutaneous infection site to the dLNs for systemic virus spread (9, 41), we hypothesized that reduced clearance of DENV in the skin may allow enhanced trafficking of infected cells to the dLNs in CD1d-KO mice. To investigate whether this occurred, we injected CFSE into the FPs of WT and CD1d-KO mice 3 days after s.c. DENV2 infection and tracked CFSE^+^ and CFSE^–^ monocyte/macrophages (CD11b^+^CD11c^–^MHC-II^+^, gating strategy, Supplemental Figure 4) and conventional DCs (cDCs, CD11c^+^CD11b^–^MHC-II^+^, gating strategy, Supplemental Figure 4) in the dLNs at day 5 after infection by flow cytometry. We detected significantly higher numbers of DENV-infected monocyte/macrophages (Figure 4G) and cDCs (Figure 4H) in the dLNs of CD1d-KO mice by intracellular staining for the DENV replication product NS3. We also observed significantly higher numbers of skin-derived (CFSE^+^) DENV-infected monocyte/macrophages (Figure 4G) and cDCs (Figure 4H) in the dLNs of CD1d-KO mice. These data indicate that the delayed viral clearance observed in the dLNs of CD1d-KO mice (Figure 4, B and C) coincides with increased migration of DENV-infected monocytes and DCs from the infected skin, where viral clearance is also impaired.

Our data also indicated that the changes in trafficking within the skin were cell-type specific, since the responses of other T cells, including γδT cells, which are important for early flavivirus clearance from skin (11, 42), were unaffected in the skin due to CD1d deficiency (Supplemental Figure 5A). While we observed a significant reduction in total CD4^+^ (Figure 4I) and γδT cell (Supplemental Figure 5B) numbers at days 3 and 5 after infection in the dLNs of CD1d-KO mice, there were no differences in the numbers of activated CD4^+^ and γδT cells between WT and CD1d-KO mice (Figure 4I and Supplemental Figure 5B). Taken together, these data demonstrate that during DENV infection, CD1d^dep^ NKT cells influence T cell recruitment in the skin and dLNs in a site-specific manner by promoting CD8^+^ CTL recruitment and activation in the skin and expansion of CD4^+^ and γδT cell populations in the dLNs without affecting their activation potential.

### CD1d^dep^/iNKT cells establish Th1 polarity during DENV infection.

**Figure 5:**
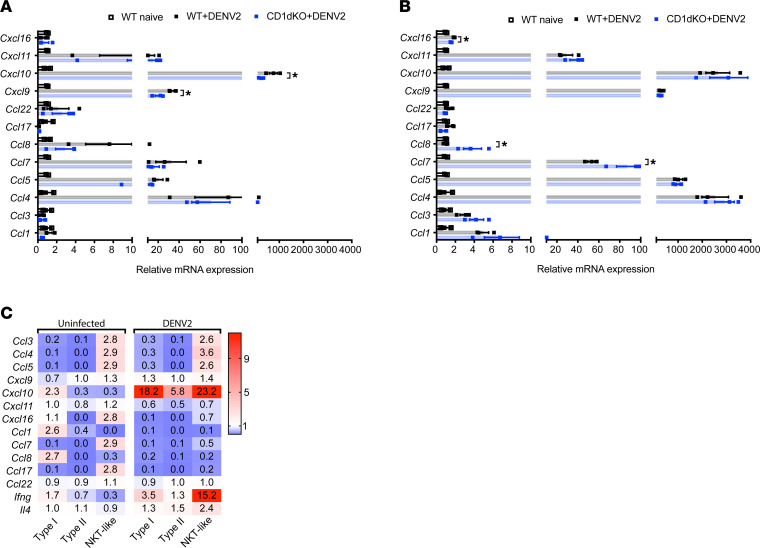
CD1d^dep^ NKT cells promote the expression of Th1-associated cytokines and chemokines in DENV-infected skin. mRNA expression levels for various Th1 and Th2 chemokines in the (**A**) FP and (**B**) dLNs of WT and CD1d-KO mice were compared 48 hours following s.c. DENV2 infection, quantified by real-time PCR (*n* = 3 biological replicates). For **A** and **B**, data are shown as means ± SEM and presented relative to WT uninfected controls. Dots represent technical replicates. *P < 0.05 between WT and CD1d-KO mice determined by 2-way ANOVA with Bonferroni’s post test. (**C**) mRNA expression levels for cytokines/chemokines were determined by real-time PCR for individual NKT cell subsets, which were sorted from dLNs harvested from naive WT mice or DENV2-infected WT mice at 48 hours after infection (for each genotype, *n* = 15 dLNs were pooled before sorting). The results are the average of triplicates, showing gene expression in types I, II, or NKT-like cells isolated from uninfected or DENV2-infected WT mice relative to expression in total NKT cells isolated from uninfected WT mice. Cytokine and chemokine responses are characteristic of a Th1 environment during DENV infection.

We hypothesized that the defects in CTL and NKT-like cell recruitment to the skin and the enhanced trafficking of infected cells from the skin to the dLNs in CD1d-KO mice could be due to altered chemotactic gradients. Therefore, we compared the mRNA expression levels of various chemokines in the FPs and dLNs of WT and CD1d-KO mice following s.c. DENV2 infection. At 48 hours after infection, significant reductions in the mRNA expression levels of *Cxcl9* and *Cxcl10* were detected in the FP skin of CD1d-KO mice compared with WT mice (Figure 5A). These chemokines are well established as important for the recruitment of Th1 cells and activated CD8^+^ T cells that express the chemokine receptor CXCR3 (43). Similarly, in the dLNs of CD1d-KO mice compared with WT mice, we detected a significant reduction in mRNA expression levels for *Cxcl16* (Figure 5B), a chemokine for CXCR6-expressing Th1 cells (44), but not *Cxcl10*. Consistent with increased infiltration of infected monocytes and DCs in the dLNs of CD1d-KO mice (Figure 4, G and H), significant increases in mRNA expression levels for *Ccl7* and *Ccl8* (Figure 5B) were detected in the dLNs of CD1d-KO mice compared with WT mice. *Ccl7* and *Ccl8* are well-established attractants for monocytes and DCs as well as some cell types with Th2 phenotypes (43, 45–50). These differences in the expression levels of various chemokines suggested a shift from a Th1 toward a Th2 bias in CD1d-KO mice in both the skin and dLN.

To identify which subsets of NKT cells produce Th1-associated chemokines, we further examined chemokine expression by each NKT cell subset following DENV infection. For this, we sorted type I/iNKT, type II, and NKT-like cells from the dLNs of DENV2-infected WT mice at 48 hours after infection and examined mRNA expression levels in each NKT cell subset for Th1 and Th2 chemokines by real-time quantitative PCR (qPCR). The fold changes in chemokine expression levels in type I/iNKT, type II, and NKT-like cells from DENV2-infected mice were compared with the fold changes in expression levels in total NKT cells from uninfected mice to indicate the relative contributions of each subset to the cytokine profile observed. In the DENV-infected WT mice, we detected approximately 18- and approximately 6-fold increases in *Cxcl10* expression in type I/iNKT and type II NKT cells relative to baseline, respectively, while *Cxcl10* expression by NKT-like cells increased approximately 23-fold (Figure 5C), indicating that NKT cells, particularly type I/iNKT and NKT-like subsets, are important producers of *Cxcl10*. Consistent with the increased expression of Th1 chemokine *Cxcl10*, *Ifng* expression was also significantly increased in type I and NKT-like subsets (Figure 5C), supporting that type I/iNKT and NKT-like cells promote a Th1 response during DENV infection.

### CD1d^dep^ NKT cell deficiency results in Th1/Th2 imbalance.

**Figure 6:**
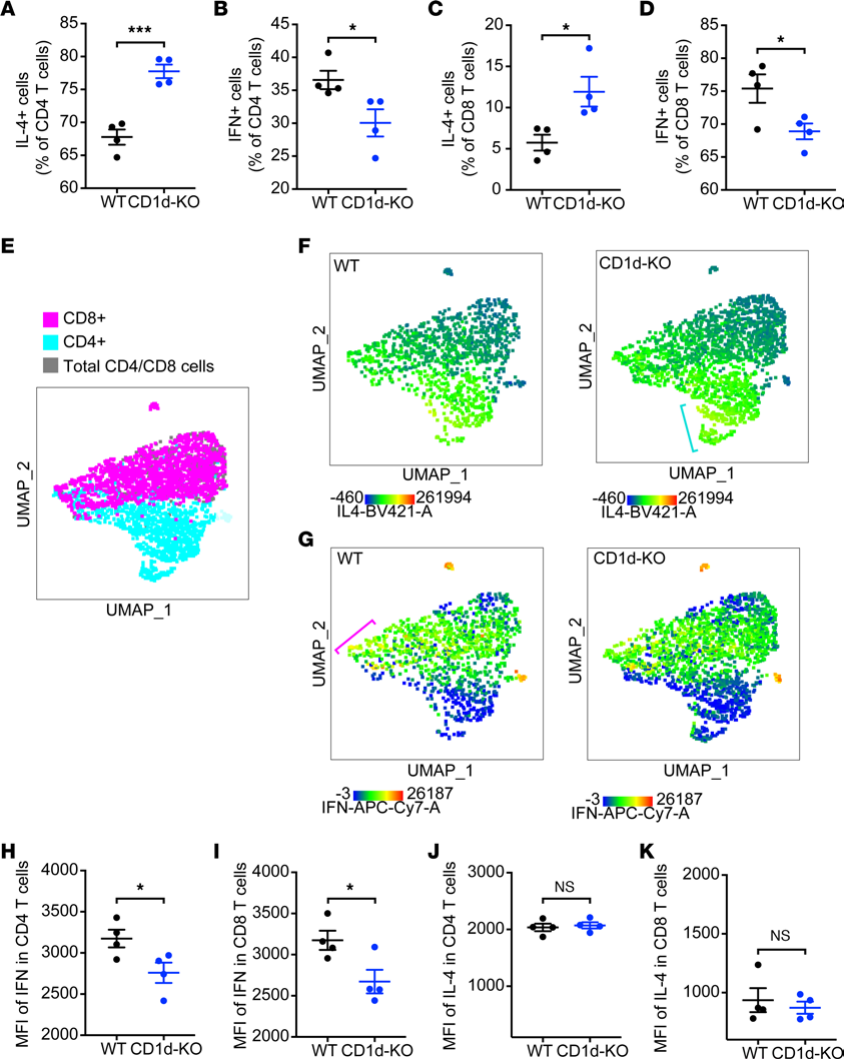
CD1d^dep^ NKT cells promote Th1-associated cellular immunity. The frequency of IL-4– or IFN-γ–producing CD4^+^ and CD8^+^ T cells in the LNs of WT and CD1d-KO mice was determined by intracellular staining, 3 days after s.c. DENV2 infection (*n* = 4). Percentages of (**A**) IL-4–producing CD4^+^ T cells (NK1.1^–^CD3^+^CD4^+^IL-4^+^), (**B**) IFN-γ–producing CD4^+^ T cells (NK1.1^–^CD3^+^CD4^+^IFN-γ^+^), (**C**) IL-4–producing CD8^+^ T cells (NK1.1^–^CD3^+^CD8^+^IL-4^+^), and (**D**) IFN-γ–producing CD8^+^ T cells (NK1.1^–^CD3^+^CD8^+^ IFN-γ^+^) are shown. (**E**) UMAP analysis showing CD4 and CD8 populations in infected dLNs. (**F**) A heatmap presentation of increased density of IL-4–expressing cells in CD1d-KO mice compared with WT in the CD4 compartment. Blue bracket indicates region of interest with increased density of IL-4^hi^ cells. (**G**) Conversely, increased intensity for IFN-γ expression in WT mice compared with CD1d-KO mice in the CD8 compartment. Pink bracket indicates region of interest with IFN-γ^hi^ cells. (**H** and **I**) MFI of IFN-γ in CD4^+^ and CD8^+^ T cells indicates increased expression of IFN-γ in both CD4 and CD8 compartments in WT but not CD1d-KO mice. (**J** and **K**) MFI of IL-4 did not differ in cell types. *n* = 4 mice for each group; error bars represent mean ± SEM. *P < 0.05; ***P < 0.001, Student’s unpaired *t* test. During DENV infection, CD1d^dep^ NKT cells are required for the optimal production of the Th1 cytokine IFN-γ by CD4^+^ and CD8^+^ T cells, and their absence causes a Th1/Th2 imbalance.

Since our chemokine expression data suggested Th2 skewing due to CD1d^dep^ NKT cell deficiency, we next aimed to confirm this functionality at the protein level and determine whether CD4^+^ and CD8^+^ T cells, which are key effectors of Th1/Th2 polarization, were also affected. For this, we compared the frequency of IL-4– or IFN-γ-producing CD4^+^ and CD8^+^ T cells in the dLNs of WT and CD1d-KO mice by intracellular staining. Consistent with a shift toward Th2 polarization in the absence of CD1d^dep^ NKT cells, significantly higher frequencies of IL-4–producing CD4^+^ T cells and lower frequencies of IFN-γ^+^ CD4 T cells were detected in the dLNs of CD1d-KO mice compared with WT mice (Figure 6, A and B). In addition, we compared the frequency of IL-4– or IFN-γ–producing CD8^+^ T cells in the dLNs and observed higher frequencies of IL-4–producing CD8^+^ T cells and lower frequencies of IFN-γ^+^CD8^+^ T cells in CD1d-KO mice compared with WT mice (Figure 6, C and D). Alternatively presented, a uniform manifold approximation and projection (UMAP) analysis allowed visualization of affected CD4 and CD8 populations in infected LNs (Figure 6E), where a heatmap presentation of IL-4 expression showed an increased density of IL-4–expressing cells in CD1d-KO mice compared with WT controls, particularly identifiable in the CD4^+^ cell region (Figure 6F), while increased intensity for IFN-γ expression was discernible in WT mice compared with CD1d-KO mice in the area of the UMAP plot corresponding to CD8^+^ T cells (Figure 6G). Assessment of IFN-γ and IL-4 MFI in CD4^+^ and CD8^+^ T cells indicated that expression of IFN-γ was increased in both CD4 and CD8 compartments in WT compared with CD1d-KO mice (Figure 6, H and I), while the MFI of IL-4 did not differ significantly (Figure 6, J and K) in spite of the expanded proportion of IL-4^+^ T cells in CD1d-KO compared with WT mice (Figure 6A). These data demonstrate that during DENV infection, CD1d^dep^ NKT cells are required for the optimal production of the Th1 cytokine IFN-γ by CD4^+^ and CD8^+^ T cells and that their absence causes a Th1/Th2 imbalance, resulting in an enhanced presence of CD4^+^ and CD8^+^ T cells producing the Th2 cytokine IL-4.

**Figure 7:**
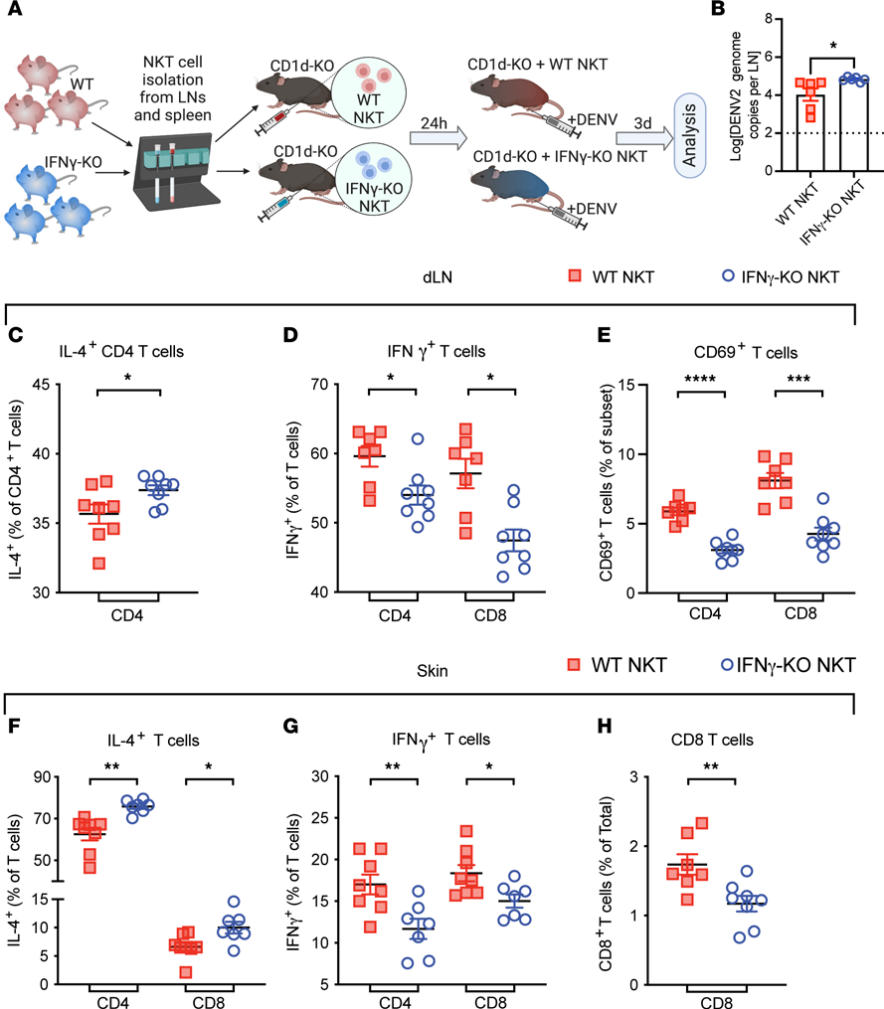
Th1 polarization is dependent on IFN-γ production by NKT cells. (**A**) A schematic for the NKT cell-transfer experiment showing purification of NKT cells from WT or IFN-γ–KO mice and adoptive transfer 1 day prior to DENV2 infection in CD1d-KO mice. (**B**) DENV genome copies in dLNs on day 3 after infection. FP skin and dLNs were collected for intracellular staining of IL-4 and IFN-γ at 3 days after infection. Frequencies of (**C**) IL-4–producing CD4^+^ and (**D**) IFN-γ–producing CD4^+^ and CD8^+^ T cells in the dLNs after infection. (**E**) Frequencies of activated CD4^+^ and CD8^+^ T cells in the dLNs after infection. Frequencies of (**F**) IL-4– and (**G**) IFN-γ–producing CD4^+^ and CD8^+^ T cells in the FP skin after infection. (**H**) Frequencies of total CD8 in the FP skin after infection. *n* = 7–8 mice per group. *P < 0.05; **P < 0.01; ***P < 0.001; ****P < 0.0001. For all panels, Student’s unpaired *t* tests were used, including for **D**–**G**, where CD4^+^ or CD8^+^ T cells were compared between groups. IFN-γ produced by NKT cells is required for optimal Th1 immunity.

Having identified defects in Th1 immunity in CD1d-KO mice and the induction of Th1 cytokine IFN-γ by iNKT and NKT-like cells in response to DENV infection (Figure 5 and Figure 6), we hypothesized that IFN-γ production by NKT cells could initiate the Th1 microenvironment. To test this, we adoptively transferred isolated NKT cells from either WT or IFN-γ–KO mice into CD1d-KO recipients prior to DENV infection (Figure 7A). At day 3 after infection, we observed increased infection in the LNs of mice that had been transferred CD1d-KO NKT cells compared with WT NKT cells (Figure 7B). We then measured cytokine production and cellular activation in the CD4^+^ and CD8^+^ T cell compartments of the skin and dLNs. NKT cell transfer from IFN-γ–deficient into CD1d-KO mice resulted in a significant increase in CD4^+^ T cell but not CD8^+^ T cell IL-4 production in the dLNs (Figure 7C and Supplemental Figure 6A) and a decrease in CD4^+^ and CD8^+^ T cell IFN-γ production during DENV infection compared with transfer of NKT cells from WT mice (Figure 7D). Transfer of WT NKT cells prior to DENV infection also promoted increased activation of CD4^+^ and CD8^+^ T cells in the dLNs compared with transfer of IFN-γ–deficient NKT cells (Figure 7E). The influence of NKT cell–derived IFN-γ on Th1 polarization was also observed in the skin site of infection (Figure 7, F and G). Consistent with the dLNs, IL-4–producing CD4^+^ and CD8^+^ T cells were fewer (Figure 7F) and IFN-γ–producing T cells were increased (Figure 7G) in the DENV-infected skin of animals adoptively transferred with WT NKT cells compared with IFN-γ–deficient NKT cells. The IFN-γ–deficient NKT cell transfer also led to reduced recruitment of CD8^+^ T cells in the skin during DENV infection, compared with transfer of WT NKT cells (Figure 7H), while the frequencies of NKT cells in the skin did not differ between the 2 groups (Supplemental Figure 6B). These data show the contribution of IFN-γ produced specifically by NKT cells in promoting the Th1-type response. In the absence of IFN-γ in NKT cells, IL-4 production by conventional T cells is enhanced.

### NKT cells promote antibody production that protects against homologous and heterologous reinfection.

**Figure 8:**
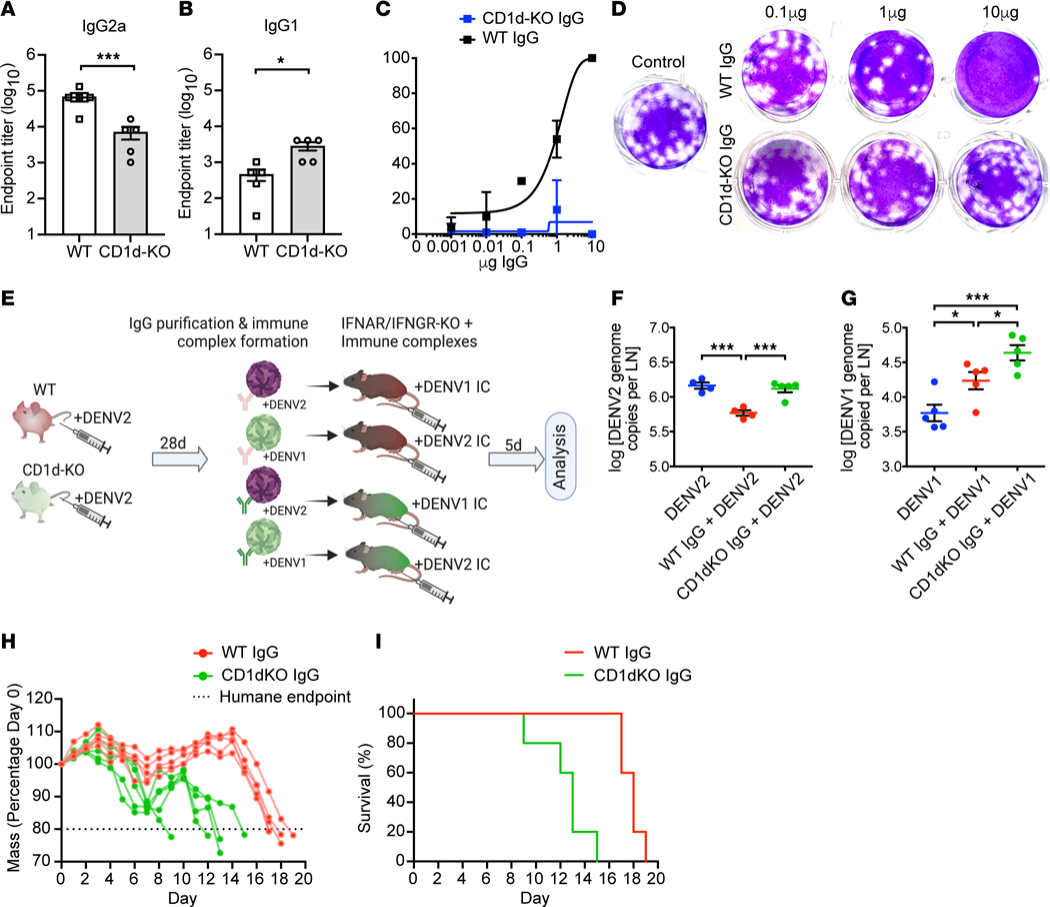
CD1d^dep^ NKT cells promote Th1-associated humoral immunity that protects during homologous and heterologous reinfections. Comparison of serum (**A**) IgG2a and (**B**) IgG1 titers between WT and CD1d-KO mice, 3 weeks following DENV2 infection, determined by ELISA. WT and CD1d-KO mice received 1 × 10^6^ PFU of DENV2 via i.p. injection. Endpoint titers were compared by Student’s unpaired *t* test. *P < 0.05; ***P < 0.001. (**C** and **D**) Plaque reduction neutralization test was performed using multiple equivalent concentrations of IgG purified from the serum of WT and CD1d-KO mice. Representative images are shown in **D**. (**E**) Schematic diagram of IgG purification from DENV2-immune WT or CD1d-KO mice, followed by immune complex formation with either DENV1 or DENV2 and injection into recipient IFN-αR/IFN-γR-KO mice. (**F**) Mice were infected with 1 × 10^5^ PFU of DENV2 or immune complexes formed from 1 × 10^5^ PFU of DENV2 and 1 μg of IgG purified from DENV2 postimmune serum from either WT or CD1d-KO mice (homologous challenge). (**G**) Mice were infected with 2 × 10^5^ PFU of DENV1 or immune complexes formed from 2 × 10^5^ PFU of DENV1 and 10 μg of purified IgG from DENV2 postimmune serum from either WT or CD1d-KO (heterologous challenge). For **F** and **G**, DENV virus burden was detected by PCR 5 days after infection. Data are represented as means ± SEM. *P < 0.05; ***P < 0.001, 1-way ANOVA with Holm-Šidák post test. (**H**) Weight loss was significantly more severe in IFN-αR/IFN-γR–KO mouse transferred antibodies from CD1d-KO DENV2-immune mice compared with those transferred antibodies from WT DENV2-immune mice following DENV1 challenge. Because of missing data resulting from lack of survival, curves were compared by restricted maximum likelihood (REML) mixed effects model and *P* < 0.0001. (**I**) Survival also differed significantly in IFN-αR/IFN-γR–KO mice by log-rank test. For **A**–**C** and **G**–**I**, *n* = 5 per group. For **H**, *n* = 4–5 per group.

Since the Th1/Th2 cytokine imbalance could directly influence humoral immunity (51) and based on our observations linking antibody subclasses and NKT cell biomarkers in humans (Figure 1), we further examined how CD1d^dep^ NKT cell deficiency affects the Th1/Th2 balance in the antibody response against DENV. For this, we infected WT and CD1d-KO mice with DENV2 and determined DENV2-specific serum IgG2a (Th1-associated) and IgG1 (Th2-associated) (51–53) antibody titers at 5 weeks after infection. Although total antibody titers against DENV determined by ELISA did not differ (Supplemental Figure 7), we observed significantly higher DENV-specific IgG2a titers in WT mice compared with CD1d-KO mice (Figure 8A), while IgG1 titers were higher in CD1d-KO mice (Figure 8B). These changes indicate a strong Th1 antibody response against DENV2 in WT mice and a Th2 skewing of the antibody response against DENV2 in CD1d-KO mice. Assessment of the neutralizing capacity of the purified IgG from WT and CD1d-KO mice also indicated that IgG from CD1d-KO mice was unable to neutralize DENV2 efficiently (Figure 8, C and D), supporting the role of CD1d^dep^ NKT cells on antibody function and raising the further question of how the persistence of a Th2-skewed profile could influence subsequent immunity to DENV.

Given the differential protective responses during homologous and heterologous secondary infections with dengue, we tested the influence of antibodies produced in WT or CD1d-KO mice with both types of challenge. For this, we purified serum IgGs from naive WT, DENV2-immune WT, and DENV2-immune CD1d-KO mice 28 days after infection prior to adoptive antibody transfer and challenge of recipients (Figure 8E). Prior to transfer of antibodies, immune complexes were formed using either purified IgGs and DENV2 (homologous challenge) or purified IgGs and DENV1 (heterologous challenge). The immune complexes were then injected into the skin of IFN-αR/IFN-γR–KO mice, which are highly susceptible to DENV infection (54), and viral titers were measured in the dLNs at 5 days after infection. In the homologous challenge model, IgGs from DENV2-immune WT mice provided protection against DENV2 infection, resulting in reduced viral titers compared with the IgGs from naive or CD1d-KO infection groups (Figure 8F). In contrast, with heterologous challenge, IgGs from DENV2-immune WT mice increased DENV1 infection, consistent with ADE (Figure 8G). However, ADE of DENV1 was significantly enhanced in the presence of IgGs from DENV2-immune CD1d-KO mice compared with IgGs from DENV2-immune WT mice (Figure 8G). Next, we questioned whether the ADE observed with IgG from CD1d-KO mice could worsen dengue disease. Using the heterologous infection model described above, we measured body mass and survival in IFN-αR/IFN-γR–KO mice. DENV1 infection in mice who received IgG from DENV2-immune CD1d-KO mice showed significantly more weight loss (Figure 8H) and higher mortality (Figure 8I) compared with the mice who received IgG from DENV2-immune WT mice. These results were also consistent using an immunocompetent model of dengue infection where weight loss was increased in mice given CD1d-KO immune complexes, although both groups recovered (Supplemental Figure 8). This supports that a higher anti-DENV IgG1/IgG2a ratio may promote the development of ADE upon subsequent heterotypic DENV infection. Therefore, CD1d^dep^ NKT cell regulation of Th1 immune polarization during DENV infection not only enhances cellular immunity and recruitment to assist with DENV infection clearance, but also alters the long-term immune status toward a protective Th1 serological profile.

### Association of preexisting Th2-associated IgG4 with dengue severity in humans.

**Figure 9:**
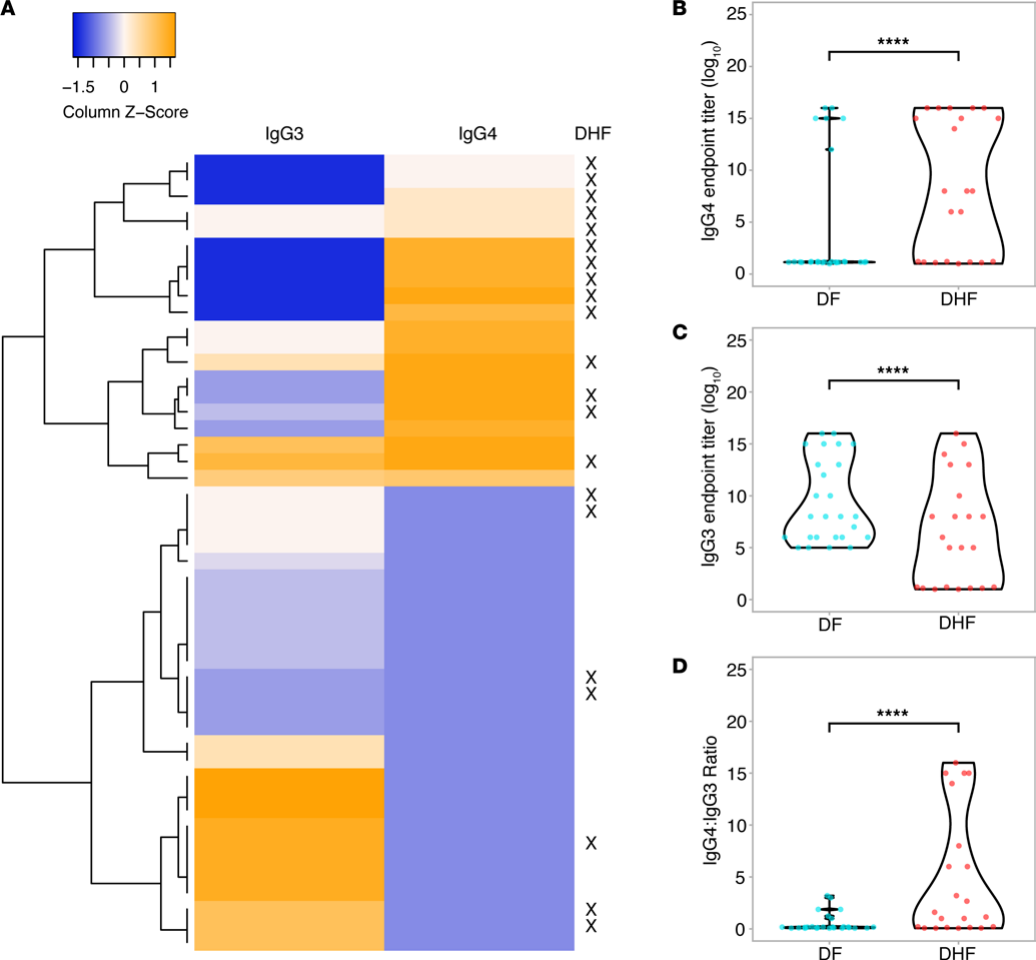
Positive association between IgG4 induction and dengue severity in humans. (**A**) A heatmap representing the DENV-specific IgG3 and IgG4 levels in pediatric dengue patients with secondary DENV infections. Patients diagnosed with DHF are indicated by an X to the right of the heatmap. (**B**) IgG4 and (**C**) IgG3 endpoint titers in the serum of secondary dengue patients who were diagnosed with DF or DHF, measured in the serum isolated on days 6 to 7 after fever onset. (**D**) Ratio of DENV-specific IgG4 to IgG3. For panels **B**–**D**, endpoint values were compared between groups by Student’s unpaired *t* test. ****P < 0.001. For all panels, *n* = 26 (DF) and *n* = 22 (DHF) patients.

Next, we evaluated whether Th2-polarized antibody responses are associated with severe disease in humans. For this, we measured DENV-specific IgG1, IgG2, IgG3, and IgG4 in a cohort of secondary dengue patients. Interestingly, patients who developed dengue fever (DF), the mild form of dengue disease, had higher levels of IgG3 (a Th1-associated antibody) and lower levels of IgG4 (a Th2-associated antibody) compared with those who developed dengue hemorrhagic fever (DHF), a severe form of dengue (Figure 9, A–C). Levels of DENV-specific IgG1 did not differ between mild and severe patients, while IgG2a was only detected in a small number of patients (Supplemental Figure 9). Consistent with this, the ratio of IgG4/IgG3 was significantly higher in DHF patients compared with DF patients (Figure 9D). This further supports our mechanistic data showing that Th2 skewed antibodies that are formed in the absence of NKT cell functionality are weakly neutralizing and may promote antibody-enhanced disease severity.

## Discussion

A first exposure to a viral pathogen is typified by infection clearance through cytotoxic T cell responses, including those of innate-like T cells (55). However, it has been unclear whether innate-like T cell functionality during a primary infection can influence disease outcomes during a secondary reexposure. It is also unclear how innate-like T cells, such as NKT cells, could function to bridge innate and adaptive immune responses in these contexts in ways independent of their own capacity to develop a memory phenotype. Here, we showed that NKT cells do not merely promote an acute antiviral response through cytotoxicity, but also help guide the fate of the memory lymphocyte pool during a primary adaptive immune response, in ways that can be protective much later during reinfection.

By using mouse models of CD1d deficiency, we showed that IFN-γ derived specifically from CD1d^dep^ NKT cells during primary DENV infection was instrumental in defining infection outcomes during a secondary DENV infection. In humans, a cytokine signature involving early induction of granzyme K in the acute phase of primary dengue disease was strongly linked to induction of Th1-associated IgG3 as infection resolved. Although granzyme K is not specific to NKT cells (32), given its importance to their function, we investigated the contributions of NKT cells in immune polarization and infection clearance in mice. Mechanistically, CD1d^dep^ NKT cells established IFN-γ–driven Th1 bias that extended to T cell responses as well as to antibody subclasses and their functionalities, which paralleled our observations in primary dengue patients. In particular, DENV-specific antibodies evoked in the absence of CD1d^dep^ NKT cells were poorly neutralizing in spite of having been generated in the presence of a higher infection burden. Functionally, these antibodies were enhancing for viral replication during secondary heterologous DENV infection and even failed to protect in a homologous DENV challenge. In humans with secondary dengue infections, we also observed a correlation between the presence of DENV-specific, Th2-associated antibody subclasses and a high Th2/Th1 antibody ratio with severe dengue. However, we did not have sufficient serum from this study to perform neutralization tests against all of the serotypes of DENV in circulation in Sri Lanka, so we do not know if the neutralizing capacity of serum antibodies is also linked to severe disease in this cohort. Interestingly, IgG3 antibodies have been characterized as having greater neutralizing capacity in some contexts, such as against SARS-CoV-2 virus (56). Multiple DENV infections are likely in dengue-endemic regions, and reexposure to a heterologous dengue serotype is a known risk factor for developing severe dengue (4). This emphasizes the potential that the innate immune activation profile an individual mounts during primary infection has to drive the responses that determine disease severity during secondary infection, likely many years later.

NKT cells display plasticity in their phenotypes and can secrete Th1, Th2, or Th17 cytokines under different circumstances. Most literature examining the role of NKT cell polarization has focused on the phenotypes of those cells during autoimmunity (57). Even the timing and circumstances of αGalCer administration can lead to altered Th1/Th17 versus Th2 bias (58). We observed in mice that type I/iNKT cells and NKT-like cell subsets are the sources of IFN-γ during acute dengue infection that initiate the Th1 microenvironment to which other cells, such as T cells, also contribute. TCR sequencing supported our flow cytometry data showing that CD1d^dep^ NKT cells expand the population of NKT-like cells at infection sites and dLNs and promote their cytokine production as well, considering that the presence of CD1d^dep^ NKT cells leads to increased diversity of NKT-like cell clones. It would be interesting, in future studies, to identify the infection-associated products that these diverse NKT cell clones recognize.

Evidence supports that IFN-γ is protective during dengue. For example, in one cohort, sustained IFN-γ production correlated with protection against fever and viremia (59). Furthermore, IFN-γ mediates nitric oxide production and reduces DENV replication and disease severity in mice (60, 61). Despite these and additional numerous examples of the innate protective functions of IFN-γ (62), the influence of IFN-γ on antibody quality during viral infections is not straightforward. On the one hand, IFN-γ is produced abundantly by conventional αβT cells (63) and it can promote T helper cell function (64) and B cell activation and proliferation (65). Also, IFN-γ has been shown to promote germinal center functions, particularly in the context of autoimmunity (66, 67). However, in some models of viral infections, IFN-γ is dispensable to antibody production or antibody-mediated protection from heterotypic strains (68, 69). We also previously observed that IFN-γ receptor–deficient mice have severely compromised survival compared with IFN-γ receptor–sufficient littermates, in a model of maternal antibody-mediated severe DENV infection (that is also type I interferon deficient) (70). In contrast to those systems, we see here that IFN-γ from NKT cells is protective during primary and secondary DENV infection. Transfer of NKT cells from WT mice but not IFN-γ–KO mice resulted in the restoration of a Th1-polarized immune response at the site of infection in the skin as well as dLNs, emphasizing the mechanistic importance of NKT cell–derived IFN-γ in establishing the polarization of the immune microenvironment, a role that might not have been apparent in systems with global deficiency or antibody blockade. Importantly, since IFN-γ–competent NKT cells from WT mice were sufficient to repair a Th1-polarized response, even in CD1d-KO mice, this supports the idea that the contributions of CD1d^dep^ NKT cells to Th1 polarization are independent of the ability of the host to present antigen via CD1d itself. This contrasts with previously described influences of NKT cells on B cell responses, which were CD1d^dep^ (71) or, in other models of primary viral infection, IL-4 dependent (72). These observations also demonstrate the pleiotropic nature of IFN-γ and emphasize that IFNs derived from different cellular sources or from different time points during the course of infection have unique potential to dictate disease outcomes. These outcomes also may be distinctive in hosts experiencing multiple pathogen exposures. Although humans with DENV infection have activated circulating NKT cells (73), we cannot be sure that the same functional effects of type I/iNKT cells we defined in mice translate directly to humans, particularly given that humans have substantially fewer NKT cells in the blood than mice (74, 75).

Th1/Th2 bias also influences lymphocyte trafficking, which appears to contribute to viral clearance during acute primary infection in our model. In CD1d-KO mice, chemokines important for cytotoxic cell recruitment were reduced (such as CXCL9 and CXCL10 in the FP) in addition to the broad Th1 to Th2 shifted chemokine response in both the skin and dLNs. Consistent with this, CD1d-KO mice had defects in the trafficking, proliferation, and activation of NKT-like cells and CD8^+^ CTLs into the cutaneous infection site. CFSE tracking experiments also suggested increased trafficking of infected cells from skin to the dLN, and imaging revealed greatly enhanced numbers of infected myeloid lineage cells, which are known DENV infection targets (4). Tracking of DENV-infected skin-derived monocyte/macrophage phenotype cells and DCs after infection showed that many of the infected cells in the dLNs were derived from the FP skin. Importantly, in addition to an overall higher burden of infection, CD1d-KO mice also had increased numbers of skin-derived infected cells in the dLNs compared with WT mice. This could point to impaired clearance by cytotoxic cells of the infected cells in the skin that (by nature of being primarily APCs) migrate to the dLN. Yet it is also likely that trafficking of infected cells was enhanced by the altered chemotactic response. Particularly in dLNs, enhanced production of *Ccl8* and *Ccl7* was observed in CD1d-KO mice compared with WT mice, which, being monocyte and DC chemoattractants (47), could enhance the trafficking of those cells infected already as well as potential infection targets. Thus, impaired trafficking of cytotoxic cells is coupled with enhanced trafficking of infected cells and infection targets to the dLNs in the context of CD1d deficiency. These data provide greater context for the multiple roles of NKT cells during infection. In addition to promoting innate and adaptive antiviral responses, as discussed above, they can also regulate the cellular trafficking dynamics of immune cells responsible for infection clearance and/or viral dissemination.

Previously, the influence of NKT cells on DENV infection and immunity had not been determined, especially as it pertains to uncoupling of their cytotoxic function from that of their influence on immune polarity and antibody functions. Dengue patients with severe disease were observed to have higher levels of invariant NKT cell activation than patients with mild disease (76), but this was studied only in secondary dengue patients, and whether increased NKT cell numbers resulted from activation during severe disease or was the cause of severe disease is unclear. Interestingly, that study also observed that cytokines were produced by invariant NKT cells with a higher IFN-γ/IL-4 ratio in patients with mild disease compared with those with severe disease (76). This is consistent with our results showing that Th1-associated NKT cell responses are protective. Beyond DENV, many human viral pathogens have evolved into antigenically similar, but evolutionarily divergent, families of viruses that confound or evade host defenses, for example, influenza viruses and coronaviruses (77, 78). In those contexts, insufficient protection can result in ADE of disease or breakthrough infections caused by heterotypic strains. Improved vaccination strategies to achieve strong crossprotective immune protection from subsequent challenges are needed. Our results affirm that the contributions of NKT cells to protection from viral infection extends beyond their innate functions to having a role in establishing a polarized and specific immune memory response. This may have applications for improving vaccines to allow our immune systems to respond more efficiently to heterologous sequential viral challenges.

## Methods

### Sex as a biological variable.

For human studies, data are derived from both men and women. For mouse studies, mostly female mice were used, and it is not known whether the mouse model results pertain to male mice.

### Animal studies and infections.

CD1d-KO, IFN-αR/IFN-γR–KO, and IFN-γ–KO mice were purchased from Jackson Laboratories and bred in-house at the Duke-NUS vivarium. WT mice (C57BL/6 background) were purchased from InVivos. DENV2 infection was introduced by FP injection of 2 × 10^5^ PFU or i.p. injection of 1 × 10^6^ PFU of DENV2.

### Statistics.

For comparisons of multiple groups, either 1-way or 2-way ANOVA was performed using Bonferroni’s multiple-comparison post test to determine statistical significance among groups. Unpaired Student’s *t* test was used to evaluate differences between 2 groups. Data were considered significant at *P* ≤ 0.05. All error bars represent the SEM. Multiplex cytokine data were analyzed using the data analysis software suite LEGENDplex, version 8.0, from BioLegend.

### Study approval.

All animal experiments were performed according to protocols approved by the SingHealth Institutional Animal Care and Use Committee. IRB approvals were obtained from the University of Colombo (no. EC-14-136), the National University of Singapore (no. LN-18-036E), and Nanyang Technological University (no. IRB-2014-06-016). Subjects’ guardians provided written, informed consent prior to participation in the study.

### Data availability.

Values for all data points in graphs are reported in the Supporting Data Values file. Other data are available upon request.

For additional methods, see Supplemental Methods, including Supplemental Table 1.

## Author contributions

This study was conceived by ALS and APSR, with experimental design and data interpretation by ALS, APSR, YC, and WAAS. The human biomarker study was planned by AWS, MS, and ALS, with MS overseeing patient recruitment. Experiments were conducted and data acquired by YC, WAAS, AO, and APSR. Data were analyzed by YC, WAAS, APSR, and ALS. The manuscript was written by ALS, APSR, WAAS, and YC.

## Supplementary Material

Supplemental data

Supporting data values

## Version 1 08/01/2024

In-Press Preview

## Version 2 09/17/2024

Electronic publication
