# Supplementary material for: NKT cells promote Th1 immune bias to dengue virus that governs long-term protective antibody dynamics
Source: J Clin Invest. 2024 Aug 1;134(18):e169251. doi: 10.1172/JCI169251 (PMC11405039; doi:10.1172/JCI169251)
Supplement: Supplemental data [file jci-134-169251-s019.pdf]

Supplementary materials for:

NKT cells promote Th1 immune bias to dengue virus that governs long term protective antibody dynamics

Supplementary Methods

Virus strains

For infections, clinical isolates of DENV2 (Eden2) or DENV1 (Eden1) were used. These low-passage clinical isolates were obtained from the Duke-NUS reference laboratory and derived from the clinical study: Early Dengue Infection and Outcomes Study, Eden(1). Virus strains were propagated in *Aedes albopictus* C6/36 mosquito cells (CRL-1660; ATCC), maintained in RPMI medium 1640 with 25mM HEPES, and titered using a standard plaque assay(2). They have previously been described to be infectious in WT mice(3, 4).

In vivo CFSE labeling

For administration of 5- and 6-carboxyfluorescein diacetate succinimidyl ester (CFSE), 50μM CFSE was injected into the FPs of WT or CD1d-KO mice 4 hours prior to FP DENV2 infection. FPs from the infected animals were harvested on day 3 post-infection to examine CFSE$^+$ or CFSE$^-$ NKT-like cell and CD8$^+$ T cell populations by flow cytometry. WT or CD1d-KO mice were infected via FP with DENV2 followed by FP injection of 50μM CFSE at day 3 post-infection. Subsequently, popliteal dLNs were harvested at day 5 post-infection to determine DENV2-infected CFSE$^+$ or CFSE$^-$ monocyte and DC populations by flow cytometry.

Virus and cytokine RNA quantification from tissues

DENV genome copies were measured in the FPs and dLNs, following DENV2 infection by FP injection by quantitative RT-PCR. FPs and popliteal dLNs were harvested at days 3 and 5 post-infection and homogenized in tissue lysis buffer (Buffer RLT; Qiagen) with ceramic beads (Glen Mills) using a mechanical homogenizer. Total RNA then was isolated with an RNeasy kit (Qiagen) according to the manufacturer's protocol. qRT-PCR to detect DENV2 was performed using the SuperScript III Platinum One-Step qRT-PCR kit (Invitrogen; Carlsbad, CA). Reaction mixtures were scaled down from the manufacturer-recommended 50μL to 25μL per reaction. Each reaction contained 12.5μL of 2X reaction mix, 0.25μL of 50μM DENV2 forward primer (5' CAG GTT ATG GCA CTG TCA CGA T 3'), 0.25μL of 50μM DENV2 reverse primer (5' CCA TCT GCA GCA ACA CCA TCT C 3'), 0.25μL of 10μM DENV2 probe (5' CTC TCC GAG AAC AGG CCT CGA CTT CAA 3'; Hex/bhq-1), 0.5μL of SuperScript III RT/Platinum TaqMix, and 13.75μL of 1μg of RNA prepared in RNAse free water. RT-PCR reactions were performed using Bio-Rad CFX96 Real-Time PCR Detection System (Bio-Rad; Hercules, CA). Cycling conditions were the following: 50°C for 30 min; 95°C for 2 min; 45 cycles of 95°C for 15 sec, 60°C for 1 min. For quantification, a standard curve was generated with serial dilutions of a plasmid containing the DENV2 genome.

For quantification of cytokines, cDNA was synthesized from the RNA isolated from tissues with the iScript Select cDNA Synthesis Kit (Bio-Rad) to amplify RNA containing a poly(A) tail. Real-time PCR was performed with SYBR Green reagent using Bio-Rad CFX96 Real-Time PCR Detection System (Bio-Rad; Hercules, CA). The results were averaged from triplicates from multiple (n=3-5 mice) FPs and dLNs, and the gene expression of types-I, -II, or NKT-like cells from uninfected or DENV2-infected WT mice were calculated relative to the total NKT cells isolated from uninfected WT mice.

Flow cytometry and intracellular Staining

To prepare single cell suspensions, tissues were harvested, minced, and incubated in RPMI medium (Gibco) containing collagenase (Sigma) for 30min at 37°C. Single cell suspensions were prepared using a 70μm cell strainer (Falcon). Prior to antibody staining, cells were incubated with anti-CD16/32 (BD Biosciences) to block nonspecific FcγR binding.The following antibodies were used to stain cells in this study: PerCP-Cy5.5-conjugated anti-CD3e (BD Biosciences), Brilliant Violet 650-conjugated anti-CD4 (BD Biosciences), Alexa Fluor700-conjugated anti-CD8 (BD Biosciences), PE-conjugated CD11b (eBioscience), Pacific Blue-conjugated anti-CD11c (Invitrogen), BUV395-conjugated anti-CD45 (BD Biosciences), FITC-conjugated anti-CD69 (eBioscience), APC-conjugated anti-γδTCR (BioLegend), Brilliant Violet 785-conjugated anti-NK1.1 (BioLegend), PE-conjugated CD1d tetramer (NIH Tetramer Core Facility), and APC-eFluor780-conjugated anti-MHCII (I-A/I-E) (eBioscience).

For intracellular cytokine staining, cells were first incubated for 6h at 37°C in the presence of intracellular protein transporter inhibitor, monensin (eBioscience). Subsequently, cells were surface stained, fixed, and permeabilized using Intracellular Fixation & Permeabilization Buffer Set (eBioscience) according to the manufacturer's protocol. Cells were then stained for intracellular cytokines using antibodies against APC-Cy7-conjugated anti-IFNγ (BioLegend), and Brilliant Violet 421-conjugated anti-IL-4 (BioLegend). Unconjugated anti-DENV NS3 protein antibody (GeneTex) was used to identify DENV2 infected cell followed by further staining with Alexa Fluor 680-conjugated secondary antibody for detection. For the adoptive transfer experiments, the following antibodies were used concurrently: PerCP-Cy5.5-conjugated anti-CD3e (BD Biosciences), Brilliant Violet 650-conjugated anti-CD4 (BD Biosciences), Alexa Fluor700-conjugated anti-CD8 (BD Biosciences), eFluor450-conjugated anti-CD69 (eBioscience), Brilliant Violet 786-conjugated anti-NK1.1 (BD Biosciences), APC-Cy7-conjugated anti-IFNγ (BD Biosciences), and PE-conjugated anti-IL-4 (BioLegend). Cells were acquired on LSRFortessa flow cytometers (BD Bioscience) using FACSDiVa software. Data were analyzed using FlowJo software. Cell sorting of $NK1.1^+CD3^+$ cells was performed on a BD FACSAria™ II flow cytometer.

TCR Sequencing

WT or CD1d-KO mice, n=3 per group, were injected I.P. with PBS or DENV2 at 10^6 PFU. Three days post infection, mesenteric LNs and Iliac LNs were collected and pooled. LNs were made into single cell suspensions. Live/dead staining was performed with LIVE/DEAD™ Fixable Near-IR Dead Cell Stain Kit (Invitrogen™, #L34975), prior to staining with PerCP-Cy5.5-conjugated anti-mouse antibody and PE-conjugated anti-mouse NK1.1. Live $CD3^+NK1.1^+$ cells were then sorted (50,000 cells per group). Sorted cells were stored in RNAprotect cell Reagent (Qiagen, #76526) and sent to iRepertoire (Huntsville, AL) for sequencing of the TCR-V_β region. Data were analyzed using iRepetoire's software. For the diversity index, they define diversity as 100 minus the area under the curve between the percentage of total reads and the percentage of unique CDR3s, when unique CDR3s are sorted by frequency from largest to smallest.

Confocal imaging of tissues

As described above, mice (WT and CD1d-KO) were infected with DENV2 by footpad injection and at day 3 post-infection, dLNs and FP skin were harvested, fixed using paraformaldehyde, embedded in O.C.T. compound, and cryo-sectioned at 15μm thickness. The tissue sections were permeabilized using 0.3% Triton X-100 in PBS for 30 min at room temperature (R.T.) followed by an incubation with blocking buffer (0.1% Saponin+ 5% BSA in PBS or 0.3% Triton X-100+ 3% BSA in PBS) for 2 hours at room temperature. Primary antibodies used were anti-mouse NK1.1 (#MAB7614, R&D systems), anti-DENV NS3 (#GTX103347, GTX124252, GeneTex), anti-mouse CD3e (#553058, BD Biosciences), anti-mouse CD11b (#13-0112-82, #14011282, eBiosciences), anti-mouse CD11c (#553799, BD Biosciences, #13011482, eBiosciences) at dultions 1:100-400 prepared in blocking buffer and incubated overnight at 4°C. Next, sections were washed with PBS 3-4 times before incubated for 1 hour at room temperature with secondary antibodies conjugated to AlexaFluor 488 or Streptavidin 488 (#A21110/ S-11223, Molecular Probes), Cy3 (#711-166-152, Jackson ImmunoResearch), AlexaFluor 546 (#A21111, Molecular Probes), or AlexaFluor 647 (#711-606-152/ 712-606-150, Jackson ImmunoResearch), diluted (1:500-1000) in blocking buffer. Sections were washed 3-4 times using PBS before mounting using Fluoroshield mounting medium containing DAPI (Sigma-Aldrich #F6057). Images were acquired using THUNDER Imaging Systems (Leica) and colocalization images were generated using ImageJ Software. For IHC, frozen cut sections were washed in PBS, permeabilized using permeabilization buffer (0.3% Triton X-100+ 3% BSA in PBS) for 1 hour at room temperature before being incubated with a 1:200 dilution of anti-DENV NS3 antibody (GTX124252, GeneTex) prepared in permeabilization buffer, overnight at 4°C. Sections were washed 5 times for 10 min each using PBS and subsequently treated with 0.3% H_2O_2 in PBS for 10 min at room temperature followed by PBS washes, 3 times for 10 min each. Next, sections were incubated for 1h at room temperature with secondary HRP conjugated antibody (#170-6515, Bio-Rad), prepared at a dilution of 1:500 in permeabilization buffer. Slides were extensively washed using PBS and developed using DAB substrate kit (#34002, Thermo Scientific) for 10 min at room temperature followed by washes using PBS and distilled water before mounting using vectamount (vectorlabs).

NKT cell purification and adoptive transfer

Spleen and lymph nodes (Iliac, mesenteric and popliteal) were harvested from wildtype (WT) or IFNγ-KO 6 weeks old mice. The organs were incubated in collagenase at 37°C for 30 min and prepared as single cell suspensions using 70μm cell strainers (Falcon). NKT cells were isolated from the single cell suspensions using the $NK1.1^+$ iNKT cell isolation kit mouse (Miltenyibiotec, #130-096-513) according to the manufacturer recommendations. After purification, NKT cells were transferred into 6 week old CD1d-KO mice, by injecting $5x10^5$ cells per mouse, IP. One group of CD1d-KO mice received purified NKT cells from WT mice and another group of CD1d-KO mice received NKT cells purified from IFNγ-KO mice. Twenty-four hours post-transfer, both groups were infected by FP injection with $1x10^5$ PFU of DENV2, strain Eden2. Three days post-infection, FPs and popliteal dLNs were harvested for flow cytometry or qPCR analysis.

Quantification of antibody titers

ELISAs were performed following previously published protocols(5, 6) with minor modifications. In brief, black 96-well half-area microtiter plates (Costar) were coated with $1x10^5$ PFU/mL of DENV2, Eden2 strain, in carbonate buffer (15mM Na_2CO_3, 35mM $NaHCO_3$, pH 9.6) overnight at 4°C. Plates were blocked with 3% non-fat dry milk powder in PBS 0.05% Tween-20 for 2 hours at room temperature. Plates were washed and 2-fold serial dilutions, beginning at 1:128, of mouse sera in complete sample diluent (PBS, BSA 1%, nonfat dry milk 1% W/V, normal goat serum 5% V/V (Sigma-Aldrich), and tween-20 0.05% V/V) were added, and incubated 2 hours at room temperature. After washing, alkaline phosphatase–conjugated anti-mouse IgG1 or IgG2a detection antibody (BD Biosciences, #557272 and #553389, respectively) were added at a 1:8000 dilution in secondary antibody diluent (PBS, BSA 0.5%, normal goat serum 5% V/V (Sigma-Aldrich), and tween 20 0.05% V/V) and incubated for 2 hours at room temperature. After washing, plates were incubated

with Attophos® AP fluorescent substrate system (Promega) for 45 min at room temperature in the dark and then read at 440nm (excitation)/560nm (emission) using a plate reader (Spark 10M, Tecan). Serum dilutions were considered positive if the relative light units (RLUs) measured were at least two-fold higher than the value of the naïve serum at the same dilution. Similarly, for the total specific anti-DENV2 IgG titers, the serial dilution started at 1:64. The secondary antibody, goat anti-mouse IgG-AP (Southern Biotech, #10130-04), was used at a dilution of 1:10000.

ADE assays

In vivo ADE assays were performed using homologous and heterologous challenges. For the heterologous challenge, WT mice or CD1d-KO mice were infected by IP with 10^6 PFU of DENV2, Eden2 strain. After 4 weeks, the sera were collected and IgG was purified with NAb™ Protein A/G Spin Kit (ThermoScientic). IgG (1μg) from DENV2-immune WT or DENV2-immune CD1d-KO or from naive mice was incubated with with $2x10^5$ PFU of DENV1, Eden1 strain, for 1 hour at 37°C. For homologous challenge, mice were infected and IgGs were purified as above. IgG (10μg) from DENV2-immune WT, DENV2-immune CD1d-KO or from naive mice was incubated with with $1x10^5$ PFU of DENV2, Eden2 strain, for 1 hour at 37°C.

After this incubation to generate immune complexes, they were injected by FP to IFNαR/IFNγR mice (n=5 mice per group). Five days post infection, FP skin and dLN were haversted for RNA extraction with the RNaeasy Kit (Qiagen) and one step qPCR were performed with SuperScript™ III Platinium™ One-step qRT-PCR System (Invitrogen), used with either DENV2 or DENV1-specific primers and probes, as appropriate given the homologous or heterologous challenge, to quantify the viral load.

For the survival experiment, 1μg of IgG from DENV2-immune WT or DENV2-immune CD1d-KO mice were incubated with with $2x10^6$ PFU of DENV1, Eden1 strain, for 1 hour at 37°C. Mice were injected I.P. and monitored every day for duration of the experement. Humane endpoints including weight loss were used as surrogates for survival.

Human dengue biomarker assays

Serum samples from pediatric dengue patients (<12 years of age) that were recruited as previously described(7) were used for biomarker assays. In brief, confirmed dengue patients were prospectively recruited at Lady Ridgeway Hospital, Colombo, Sri Lanka upon meeting the following study criteria: having a fever of ≥ 38°C for less than 72h, hospital admission, and confirmation of DENV infection by NS1 antigen test at the time of recruitment. Longitudinal samples obtained 1-3 days, 4-5 days, and/or 6-7 days post fever onset were used as indicated in figure legends. The final DF versus DHF diagnoses were according to the 1997 WHO critera, and viral RNA quantification was performed as previously reported(7). Research was designed to be compliant with the Declaration of Helsinki. Ethical approvals were obtained from the Institutional Review Boards of Lee Kong Chian School of Medicine, Nanyang Technological University (NTU), Singapore, National University of Singapore and the Ethics Review Committee of Faculty of Medicine, University of Colombo, Sri Lanka. Written informed consent was given by a parent or guardian of the patient.

Samples were selected for biomarkers testing on the basis of having sufficient remaining volume following their use for DENV confirmation and prior research objectives to complete the assays as well as on the basis of having matched longitudinal samples for the same patients. Primary versus secondary infections were determined by Panbio Dengue IgG Capture ELISA (Catalog number: 01PE10) and Panbio Dengue IgM Capture ELISA (Catalog number: 01PE20), both from PanBio Diagnostics. Primary cases were identified as IgM+IgG− and secondary cases were identified as IgM+IgG+(8). ELISAs and biomarker assays were performed by a blinded study team member.

For cytokine profiling, serum were analysed using the Legenplex CD8/NK panel (13-plex) (Biolegend, catalog number 740267) accordingly to the manufacturer's protocol for the following analytes: IL-2, IL-4, IL-6, IL-10, IL-17A, IFN-ɣ, TNF-α, sFas, sFasL, Granzyme A, Granzyme B, perforin, and granulysin. Beads were acquired on a LSRFortessa flow cytometer (BD Bioscience) using FACSDiVa software. The concentration of granzyme K was determined using the human ELISA kit for granzyme K (Cloud-Clone Corp., catalog number SEB209Hu) accordingly to the manufacturer's instruction. The ELISA plate was read using a plate reader (Spark 10M, Tecan).

For human antibody profiling, mouse anti-human IgG1-AP, IgG2a-AP, IgG3-AP and IgG4-AP (SouthernBiotech, catalog number 9054-04, 9070-04, 9210-04 and 9200-04, respectilvely) were used to detect DENV-specific antibodies. Half area plates were coated with DENV2 Eden strain at 10^5 PFU/mL. The sera were 2-fold serial diluted starting at 1:64 for IgG1 and IgG2a and 1:32 IgG3 and IgG4. The goat anti-human IgG1-, IgG2a-, IgG3- and IgG4 AP conjugated antibodies were used for detection at a 1:2500, 1:500, 1:1000, and 1:1000 dilution respectively, and read as described above using a plate reader. Serum dilutions were considered positive if the RLUs measured were two-fold higher than the value of the naive serum at the same dilution.

Software

Prism 5 and Excel were used to determine statistical significance. Heat map in Figure 8A was made with Heatmapper software(9). Violin plots in Figures 8B-D were made with PlotsofData(10). All diagrams were made with Biorender.com.

Supplementary Figures and Table

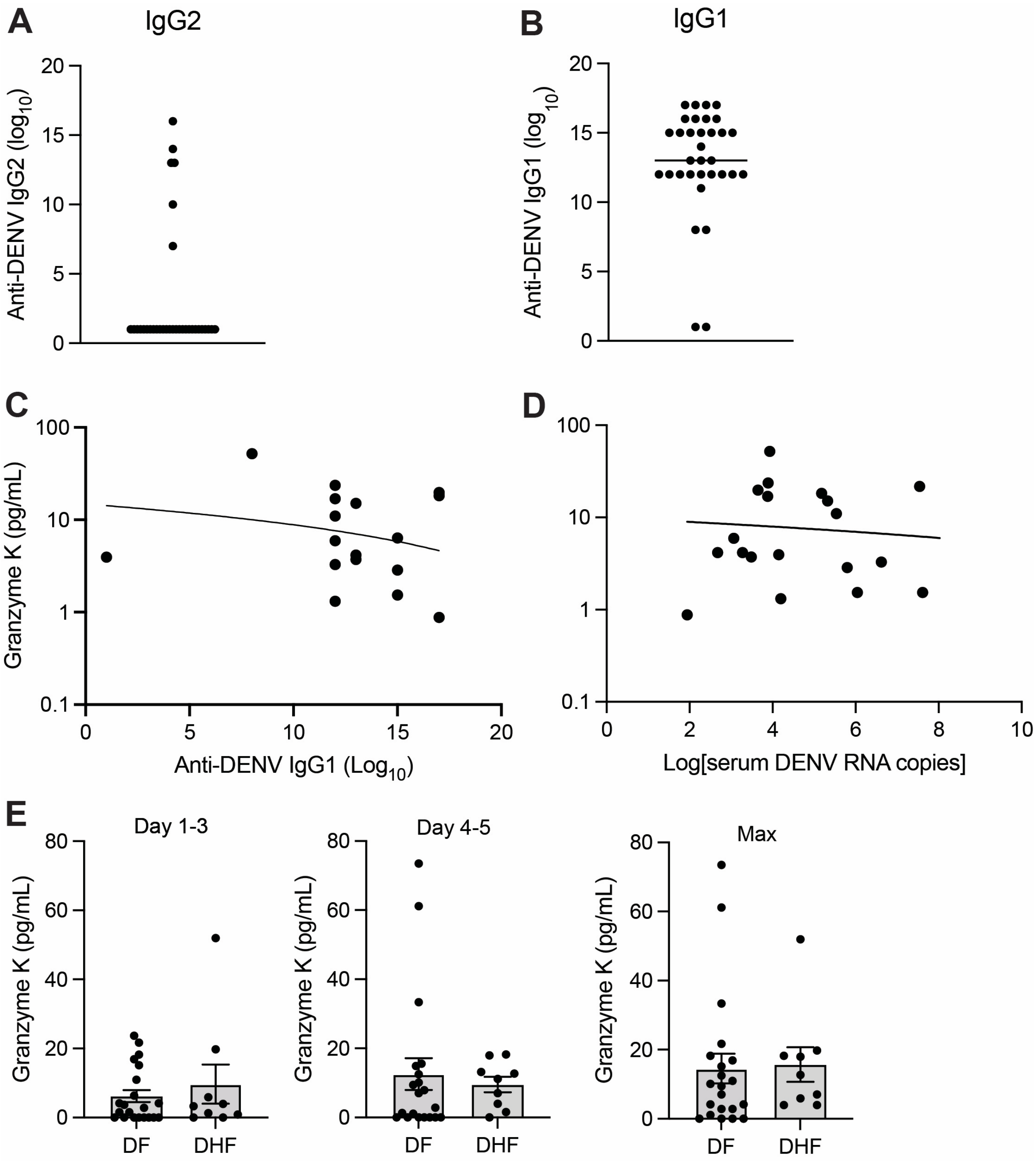

Supplementary Figure 1: No correlations between serum Granzyme K concentrations and DENV burden, disease severity or IgG1/2. (**A**) IgG2 and (**B**) IgG1 (**C**) No significant correlation between anti-DENV IgG1 titers and Granzyme K (**D**) No significant correlation between Granzyme K concentrations and serum viral burden in primary dengue patients. (**E**) No significant difference between the serum levels of Granzyme K between patients with a final diagnosis of DF versus DHF, for samples obtained days 1-3, 4-5, or the maximum value (Max) detected in either sample.

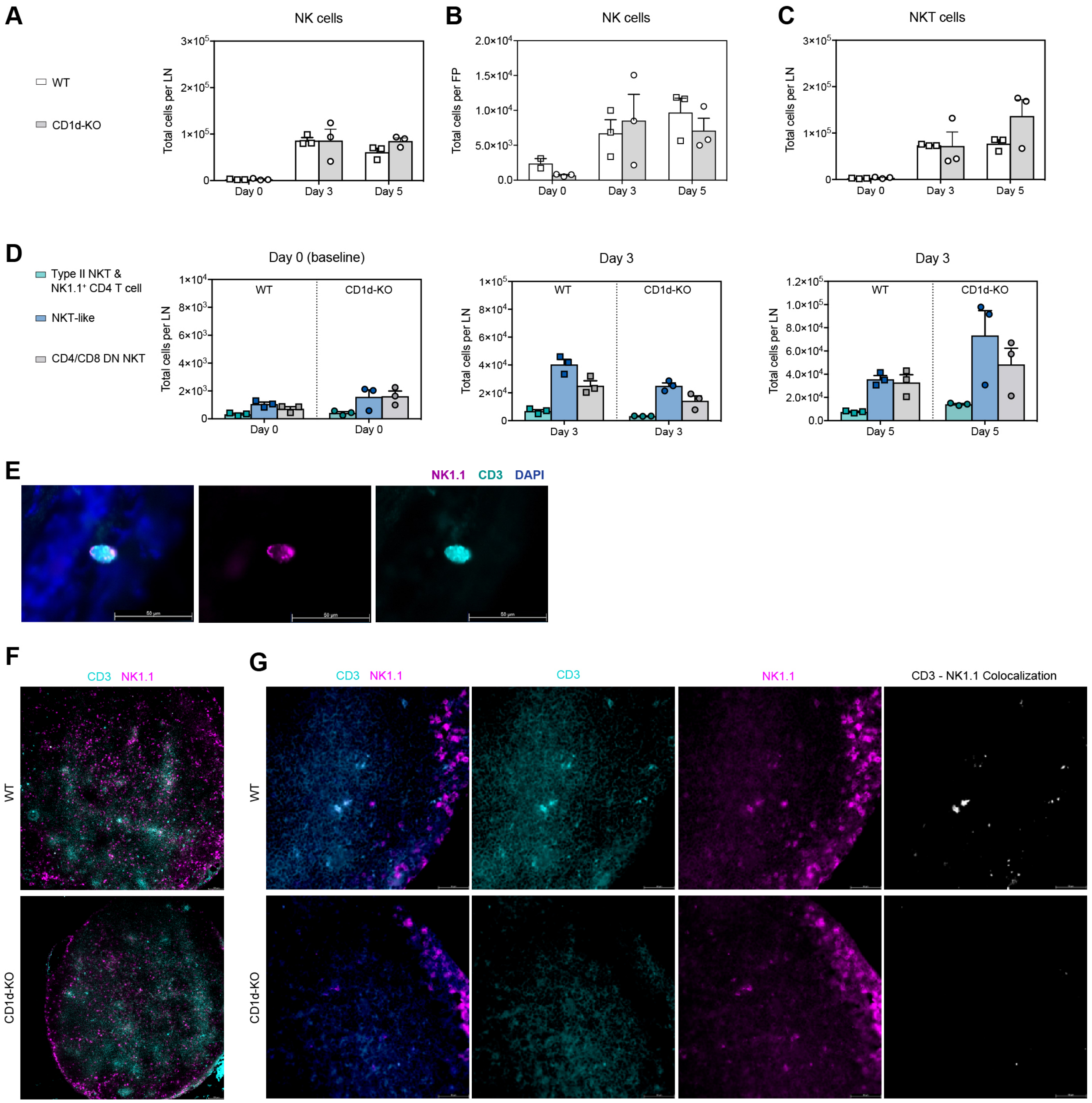

Supplementary Figure 2: Unaltered cellular parameters between WT and CD1d-KO mice following DENV infection. Quantification of NK cells in the (**A**) dLN and (**B**) FP skin and (**C**) NKT cells in the dLNs of WT and CD1d-KO mice following DENV infection. (**D**) Quantification of $CD1d^{ind}$ NKT cell subsets in the dLNs, in parallel to Figure 2F, showed no significant difference between WT and CD1d-KO mice following DENV infection. (**E**) Immunostaining of WT mice at day 3 post-infection confirmed the presence of NKT cells in the skin (image representative of n=3 mice). (**F**) Low and (**G**) High magnification confocal images of dLNs immunostained for NK1.1 and CD3, showing NKT ($NK1.1^{+}CD3^{+}$) (Images representative of n=3 per group).

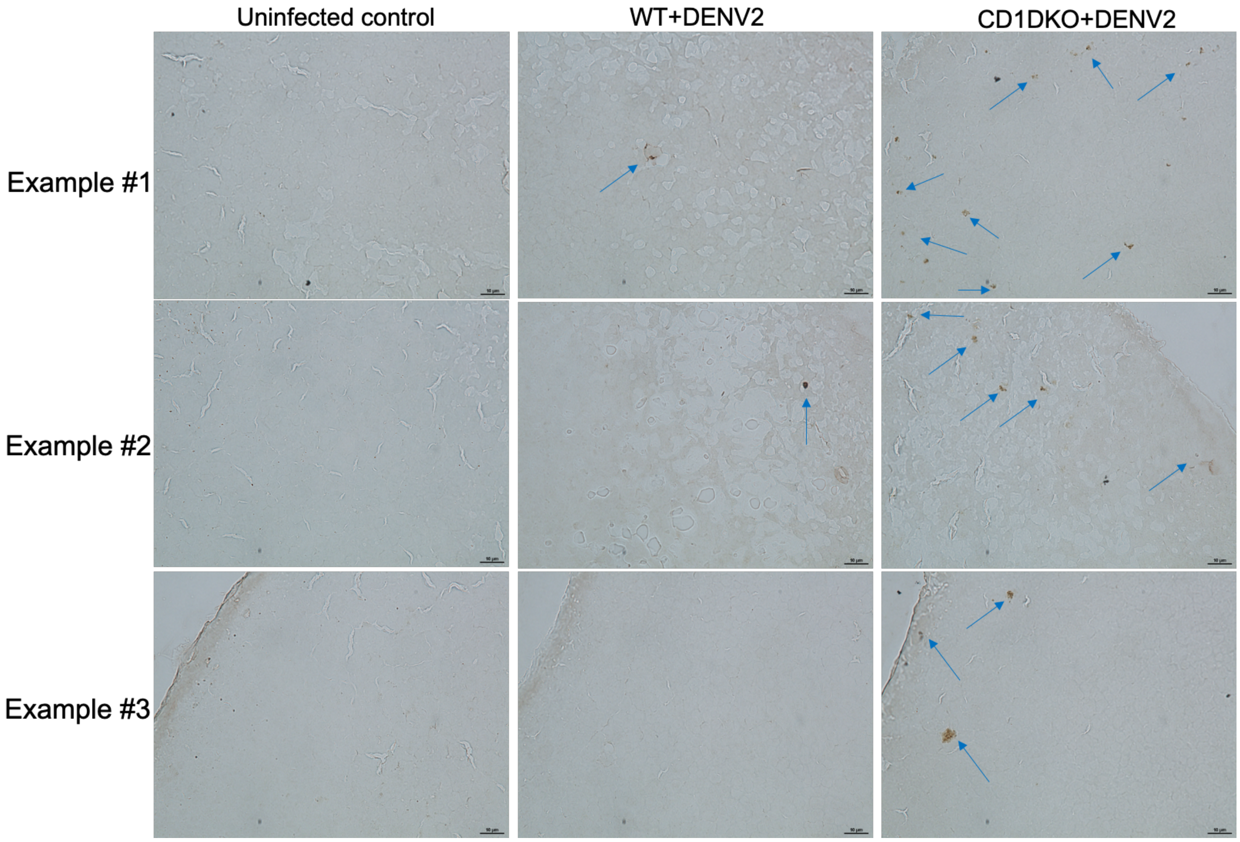

Supplementary Figure 3: Confirmation of slower DENV clearance from dLNs of CD1d-KO mice. WT or CD1d-KO mice were infected with $2x10^5$ PFU of DENV2 via F.P injection and dLNs were collected at Day 5 post-infection corresponding to the time point in Figure 4B. IHC was performed on frozen cut dLN sections and stained for DENV NS3 protein. Images show examples of increased NS3 staining (blue arrows) in the dLN of CD1d-KO mice after DENV infection compared to WT mice. The data is representative of n=2 mice, each group**.**

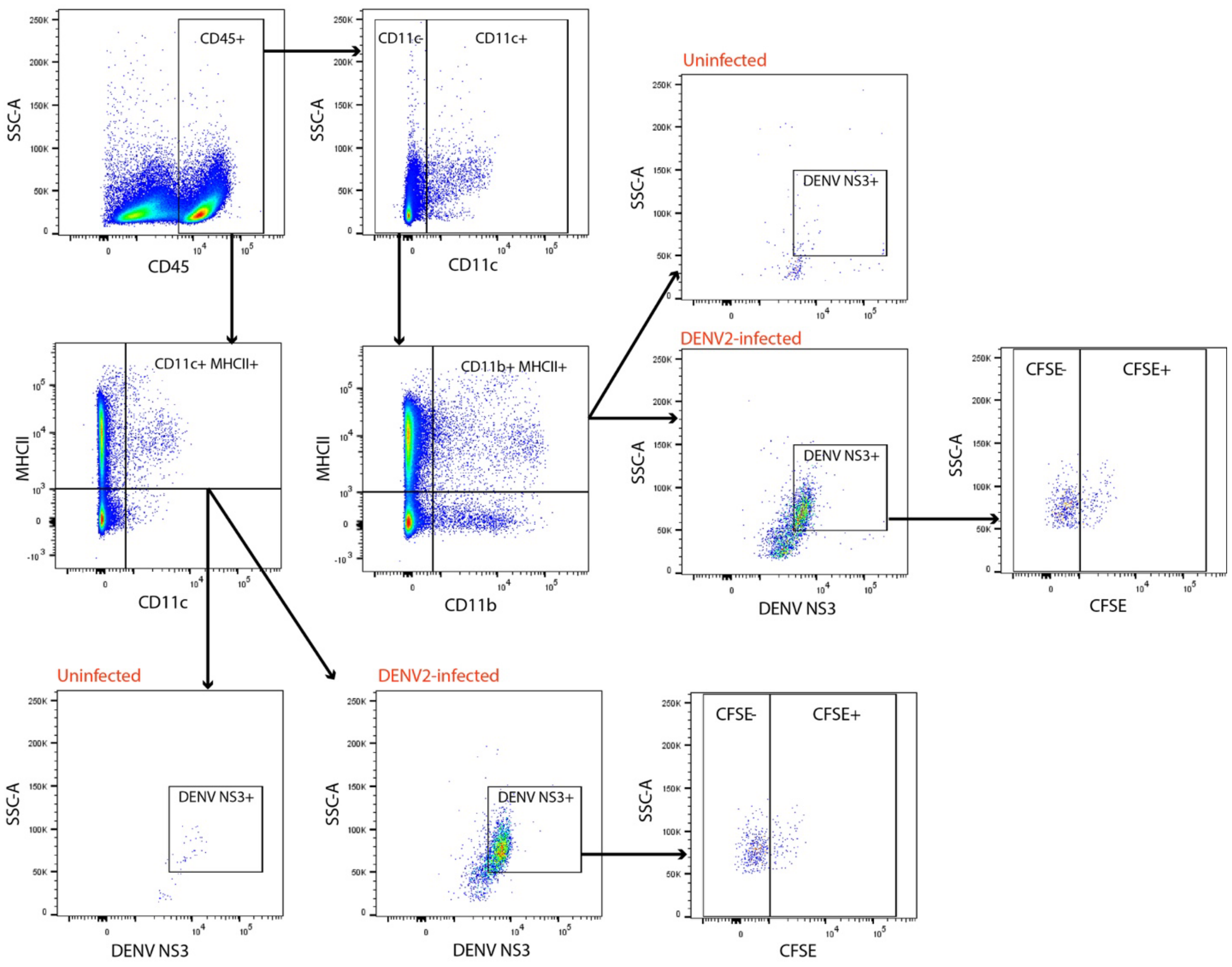

Supplementary Figure 4: Gating strategy for CFSE$^+$ or CFSE$^-$ DENV2-infected monocytes and DCs. FACS analyses for monocyte/macrophage or DC cell subsets were performed after excluding contamination of doublets by comparing forward scatter area (FSC-A) versus forward scatter height (FSC-H) profile, then comparing side scatter area (SSC-A) versus side scatter height (SSC-H) profiles. To identify monocyte/macrophages, singlets were gated for CD45$^+$ population. CD45$^+$ cells were gated for the CD11c$^-$ population to eliminiate DCs. CD45$^+$ CD11c$^-$ cells were further gated for the CD11b$^+$ and MHCII$^+$ population. DCs were identified as CD45$^+$ CD11c$^+$. Monocytes/macrophages or DCs were each then gated for the DENV NS3$^+$ population to identify DENV-infected cells which were apparent in infected but not uninfected (control) samples. DENV-infected cells were then separted into CFSE$^+$ and CFSE$^-$ populations.

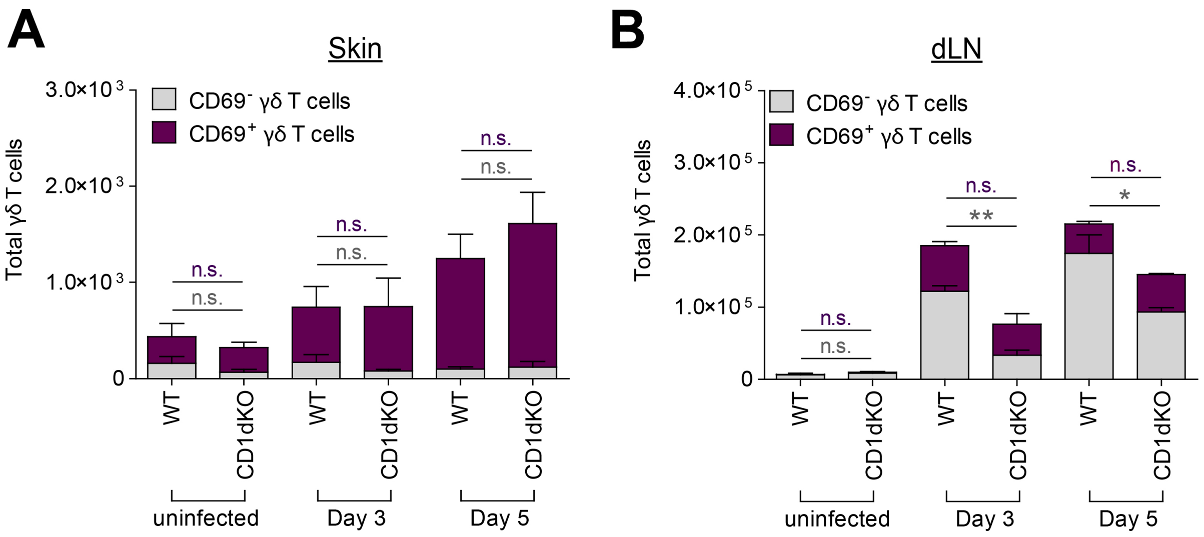

Supplementary Figure 5: No influence on γδT cell activation in CD1d-KO mice infected with DENV. Total γδT cells in the (**A**) FP and (**B**) dLN were determined on days 0, 3, and 5 post-infection. Activated CD69$^+$γδT cells are denoted in purple, CD69$^-$γδ T cells are denoted in gray, and both are presented as stacked bars. For all panels, n=6 mice for each time point. Data are shown as the mean±SEM. n.s.=not significant *p<0.05 and **p<0.01, determined by 2-way ANOVA with Tukey's posttest.

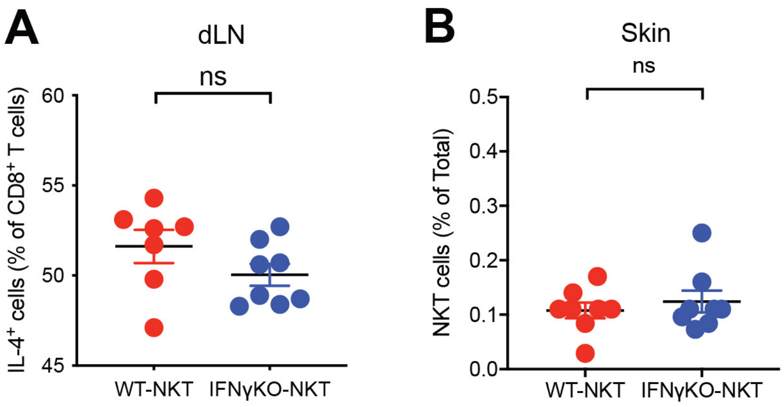

Supplementary Figure 6: Assessment of IL-4$^+$ CD8 and NKT cells in mice following adoptive T cell transfer. Percentages of (**A**) IL-4$^+$ CD8 T cells in dLNs and (**B**) NKT cells in the skin did not differ in mice following the adoptive transfer and DENV infection experiment diagramed in Figure 7A.

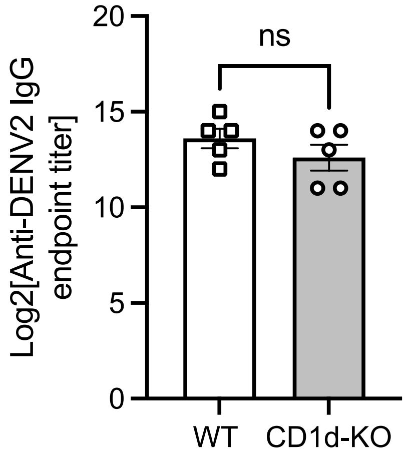

Supplementary Figure 7: Similar DENV-binding antibodies in WT and CD1d-KO mice. DENV-specific antibody binding titers determined by ELISA in serum collected 21 days post infection did not differ. Data were compared by Student's unpaired t-test, n=5.

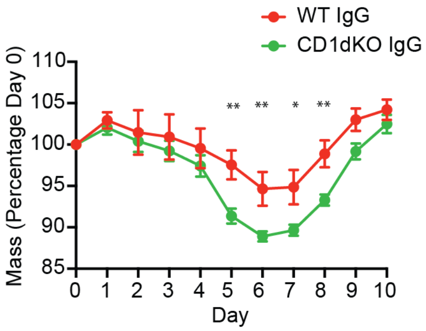

Supplementary Figure 8: Immune competent mice experience weight loss with CD1d-KO antibodies promoted DENV infection. Weight loss was significantly more severe in WT mice transferred antibodies from CD1d-KO DENV2-immune mice (according to the experimental design presented in Figure 8E) compared to those transferred antibodies from WT DENV2-immune mice following DENV1 challenge. A two-way repeated measures ANOVA was used to assess significance, with Fishers uncorrected Least Significant Difference (LSD) test used to generate p-values. *p<0.05 and **p<0.01, with n=5 mice per group.

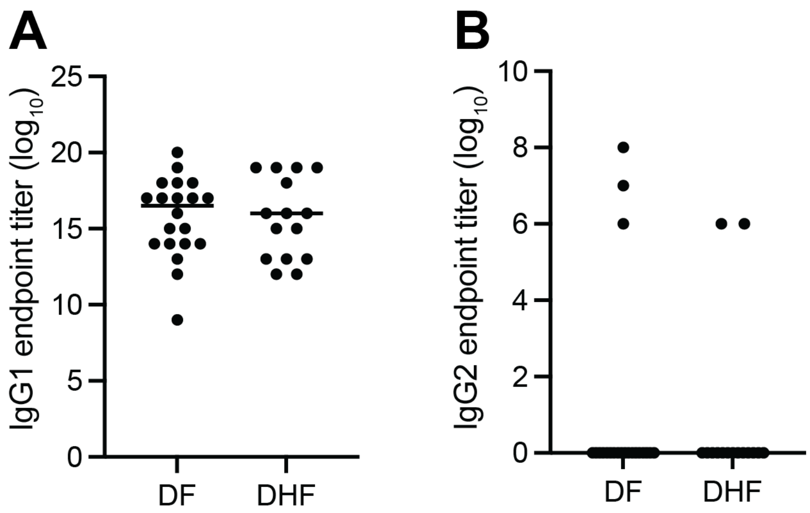

Supplementary Figure 9: Similar concentrations of IgG1 and IgG2 in the serum of human secondary dengue patients. Antibody titers were determined by ELISA in matching samples to Figure 9B-C.

Supplemental Table 1: Chemokine primer sequences

Gene	Sequence
CCL1	forward 5’ GGC TGC CGT GTG GAT ACA G 3’
	reverse 5’ AGG TGA TTT TGA ACC CAC GTT T 3’
CCL3	forward 5’ TTC TCT GTA CCA TGA CAC TCT GC 3
	reverse 5’ CGT GGA ATC TTC CGG CTG TAG 3’
CCL4	forward 5’ TTC CTG CTG TTT CTC TTA CAC CT 3’
	reverse 5’ CTG TCT GCC TCT TTT GGT CAG 3’
CCL5	forward 5’ GCT GCT TTG CCT ACC TCT CC 3’
	reverse 5’ TCG AGT GAC AAA CAC GAC TGC 3’
CCL7	forward 5’ GCT GCT TTC AGC ATC CAA GTG 3’
	reverse 5’ CCA GGG ACA CCG ACT ACT G 3’
CCL8	forward 5’ TCT ACG CAG TGC TTC TTT GCC 3’
	reverse 5’ AAG GGG GAT CTT CAG CTT TAG TA 3’
CXCL9	forward 5’ GGA GTT CGA GGA ACC CTA GTG 3’
	reverse 5’ GGG ATT TGT AGT GGA TCG TGC 3’
CXCL10	forward 5’ CCA AGT GCT GCC GTC ATT TTC 3’
	reverse 5’ GGC TCG CAG GGA TGA TTT CAA 3’
CXCL11	forward 5’ GGC TTC CTT ATG TTC AAA CAG GG 3’
	reverse 5’ GCC GTT ACT CGG GTA AAT TAC A 3’
CXCL16	forward 5’ CCT TGT CTC TTG CGT TCT TCC 3’
	reverse 5’ TCC AAA GTA CCC TGC GGT ATC 3’
CCL17	forward 5’ TAC CAT GAG GTC ACT TCA GAT GC 3’
	reverse 5’ GCA CTC TCG GCC TAC ATT GG 3’
CCL22	forward 5’ AGG TCC CTA TGG TGC CAA TGT 3’
	reverse 5’ CGG CAG GAT TTT GAG GTC CA 3’

References for supplementary materials

1. Low JG, Ooi EE, Tolfvenstam T, Leo YS, Hibberd ML, Ng LC, et al. Early Dengue infection and outcome study (EDEN) - study design and preliminary findings. *Ann Acad Med Singapore.* 2006;35(11):783-9.
2. Schulze IT, and Schlesinger RW. Plaque assay of dengue and other group B arthropod-borne viruses under methyl cellulose overlay media. *Virology.* 1963;19:40-8.
3. St John AL, Rathore AP, Raghavan B, Ng ML, and Abraham SN. Contributions of mast cells and vasoactive products, leukotrienes and chymase, to dengue virus-induced vascular leakage. *eLife.* 2013;2:e00481.
4. Rathore AP, Mantri CK, Tan MW, Shirazi R, Nishida A, Aman SA, et al. Immunological and Pathological Landscape of Dengue Serotypes 1-4 Infections in Immune-Competent Mice. *Frontiers in Immunology.* 2021;12(681950).
5. Staats HF, Nichols WG, and Palker TJ. Mucosal immunity to HIV-1: systemic and vaginal antibody responses after intranasal immunization with the HIV-1 C4/V3 peptide T1SP10 MN(A). *J Immunol.* 1996;157(1):462-72.
6. St John AL, Chan CY, Staats HF, Leong KW, and Abraham SN. Synthetic mast-cell granules as adjuvants to promote and polarize immunity in lymph nodes. *Nat Mater.* 2012;11(3):250-7.
7. Rathore APS, Senanayake M, Athapathu AS, Gunasena S, Karunaratna I, Leong WY, et al. Serum chymase levels correlate with severe dengue warning signs and clinical fluid accumulation in hospitalized pediatric patients. *Sci Rep.* 2020;10(1):11856.
8. Vaughn DW, Nisalak A, Solomon T, Kalayanarooj S, Nguyen MD, Kneen R, et al. Rapid serologic diagnosis of dengue virus infection using a commercial capture ELISA that distinguishes primary and secondary infections. *Am J Trop Med Hyg.* 1999;60(4):693-8.
9. Babicki S, Arndt D, Marcu A, Liang Y, Grant JR, Maciejewski A, et al. Heatmapper: web-enabled heat mapping for all. *Nucleic Acids Res.* 2016;44(W1):W147-53.
10. Postma M, and Goedhart J. PlotsOfData-A web app for visualizing data together with their summaries. *PLoS Biol.* 2019;17(3):e3000202.
